# Reversing insecticide resistance with allelic-drive in *Drosophila melanogaster*

**DOI:** 10.1038/s41467-021-27654-1

**Published:** 2022-01-12

**Authors:** Bhagyashree Kaduskar, Raja Babu Singh Kushwah, Ankush Auradkar, Annabel Guichard, Menglin Li, Jared B. Bennett, Alison Henrique Ferreira Julio, John M. Marshall, Craig Montell, Ethan Bier

**Affiliations:** 1 Tata Institute for Genetics and Society, Center at inStem, Bangalore, Karnataka 560065 India; 2 Section of Cell and Developmental Biology, University of California, San Diego, La Jolla, CA 92093 USA; 3 Tata Institute for Genetics and Society, University of California, San Diego, La Jolla, CA 92093 USA; 4 Neuroscience Research Institute, University of California, Santa Barbara, CA 93106 USA; 5 Department of Molecular, Cellular and Developmental Biology, University of California, Santa Barbara, CA 93106 USA; 6 Biophysics Graduate Group, Division of Biological Sciences, College of Letters and Science, University of California, Berkeley, CA 94720 USA; 7 Instituto de Ciências Biomédicas, Universidade Federal do Rio de Janeiro, Rio de Janeiro, RJ 21941-902 Brazil; 8 Division of Biostatistics and Epidemiology - School of Public Health, University of California, Berkeley, CA 94720 USA; 9 Innovative Genomics Institute, Berkeley, CA 94720 USA

**Keywords:** Genetic engineering, Eukaryote

## Abstract

A recurring target-site mutation identified in various pests and disease vectors alters the *voltage gated sodium channel* (*vgsc*) gene (often referred to as *knockdown resistance* or *kdr*) to confer resistance to commonly used insecticides, pyrethroids and DDT. The ubiquity of *kdr* mutations poses a major global threat to the continued use of insecticides as a means for vector control. In this study, we generate common *kdr* mutations in isogenic laboratory *Drosophila* strains using CRISPR/Cas9 editing. We identify differential sensitivities to permethrin and DDT versus deltamethrin among these mutants as well as contrasting physiological consequences of two different *kdr* mutations. Importantly, we apply a CRISPR-based allelic-drive to replace a resistant *kdr* mutation with a susceptible wild-type counterpart in population cages. This successful proof-of-principle opens-up numerous possibilities including targeted reversion of insecticide-resistant populations to a native susceptible state or replacement of malaria transmitting mosquitoes with those bearing naturally occurring parasite resistant alleles.

## Abstract (web summary)

Insecticide resistance (IR) poses a major global health challenge. Here, the authors generate common IR mutations in laboratory *Drosophila* strains and use a CRISPR-based allelic-drive to replace an IR allele with a susceptible wild-type counterpart, providing a potent new tool for vector control.

## Introduction

Insect-borne diseases represent a major public health concern, killing over 700,000 people annually, with children being the most vulnerable^1^. Primary efforts in limiting the spread of these diseases focus on controlling mosquito vector populations. Common vector control practices include indoor residual spraying with pyrethroids, or a few other insecticides recommended by the WHO and use of long-lasting insecticide-treated mosquito nets (LLINs), all of which incorporate a pyrethroid. Altogether, only a handful of insecticides are currently approved for use. The prolonged and repeated deployment of such a limited number of insecticides has resulted in selection for insecticide resistant (IR) insect populations, undermining ongoing global vector control efforts^1^. Mutations in insecticide-target genes are among the most common contributors to IR in numerous insect species including mosquitoes.

One solution to the growing problem of IR is to develop new insecticides, which is a focus of intensive and costly ongoing efforts^2,3^. A complementary approach is to piggyback on newly developed gene-drive technologies designed to disseminate gene cassettes through insect populations that either reduce the number of individuals (suppression drives) or modify populations so that they no longer present a threat to public health (e.g., by expressing anti-malarial effectors preventing pathogen transmission by anopheline mosquitoes)^4^. In this current study, we employ the powerful genetic tools available in the fruit fly *Drosophila melanogaster* to provide proof-of-principle for allelic-drive wherein we endow a gene-drive element with an additional functionality to bias inheritance of a preferred allelic variant (the wild-type insecticide susceptible *vgsc* allele) over a IR allele prevalent in many insects (L1014F).

## Results

### Generation of knock-down resistance alleles of the voltage-gated sodium ion channel in *Drosophila*

**Fig. 1:**
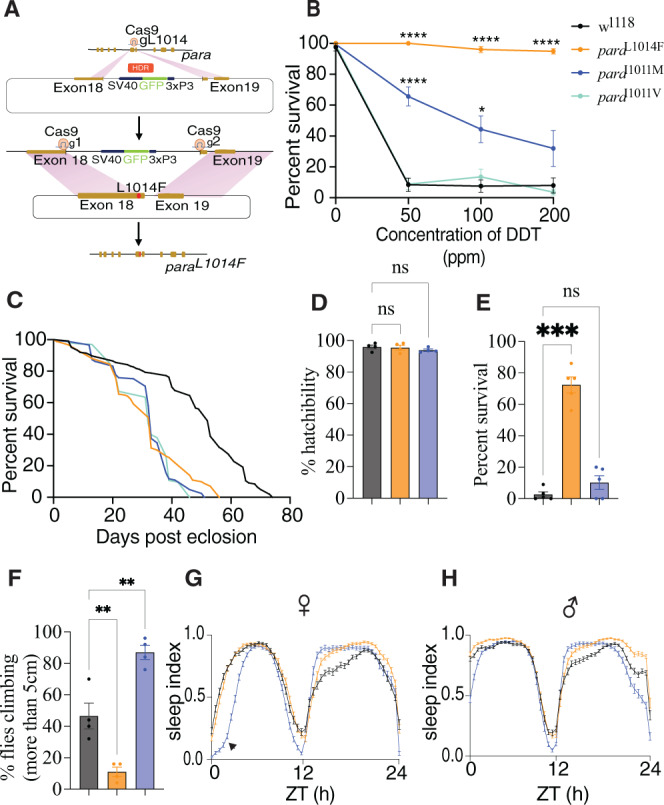
*kdr* mutations *para*^L1014F^ and *para*^I1011M^ confer insecticide resistance in *Drosophila melanogaster*. **A** Generation of *kdr* mutations. First, a GFP transgene was inserted at the L1014 site using Cas9-mediated editing. Next, the GFP insertion was deleted with a pair of gRNAs cleaving adjacent sequences and replaced with a donor template containing one of the desired mutations (L1014F, I1011M or I1011V). Successful transformants were screened as GFP negative and later confirmed by genotyping. **B** DDT resistance: Flies (total number of flies *n* = 200; number of independent replicates *N* = 8) were exposed to different concentrations of DDT. Survival, a proxy for DDT resistance, was determined 24 h post exposure. At the reference dose of 50 ppm DTT, the mean resistance values ± s.e.m were: WT = 8.4 ± 4.3; L1014F = 100 ± 0; I1011M = 65.6 ± 6.15; I1011V = 8.75 ± 3.6. **C** Survival plot for *para*^WT^ (Median lifespan = 52), *para*^L1014F^ (Median lifespan = 33), *para*^I1011M^ (Median lifespan = 33), *para*^I1011V^ (Median lifespan = 32) males in absence of any insecticide. All three mutants displayed shortened lifespans compared to the control. Data were analyzed using the Log-rank test for trend and the Mantel-Cox test. **D** Comparison of embryonic survival. No significant differences in hatching rate were observed among WT, *para*^L1014F^ and *para*^I1011M^ genotypes. *N* = 4; *n* = 400. **E** *para*^L1014F^ are resistant to oxidative stress. *para*^L1014F^ flies had significantly higher survival rates at 48 h compared to WT or to *para*^I1011M^ mutants. *N* = 5; *n* = 125. **F** *para*^L1014F^ are paralytic at high temperature. Male flies were exposed to 37 °C for 4 h and their ability to climb above 5 cm was recorded. *N* = 4; *n* = 100. **G**, **H** *para* mutants manifested abnormal sleep patterns in both females and males. Significant day-time sleep latency for *para*^I1011M^ mutants is indicated by the arrowhead. Control = Black; *para*^1014*F*^ = Orange; *para*^1011M^ = Blue; *para*^1011V^ = purple. *N*: independent biological replicates, *n*: number of animals tested; Means ± s.e.m are plotted; **B** two-way ANOVA with Sidak’s multiple comparison, **D**, **E**, **F** one-way ANOVA with Sidak’s multiple comparison. *p < 0.033, **p < 0.0021, ***p < 0.0002, ****p < 0.0001, and ns not significant.

Pyrethroids and DDT bind to the voltage-gated sodium channels (VGSCs) triggering abnormal channel activity but are unable to bind or disrupt function of Knockdown-resistant (Kdr) mutant channels. The most widespread *kdr* alleles in *Anophelines* include L1014F, whereas I1011M/V are prevalent in *Aedes aegypti*^5^. While attempts have been made to evaluate the effect of these mutations in field isolates, their direct impact on insect fitness and physiology has been challenging to assess due to genetic heterogeneity of wild insect populations^6–9^. As the protein sequences of VGSCs are highly conserved across insect orders^10^, with nearly 100% identity at the insecticide-binding site (Supplementary Fig. 1), we analyzed the effects of prevalent *kdr* field variants on insecticide susceptibility and fitness in the genetic model *Drosophila melanogaster*. As a first step toward this goal, we generated fly lines carrying mutations equivalent to L1014F, I1011M and I1011V alleles in the *Drosophila vgsc* gene ortholog *paralytic* (*para*) using CRISPR-Cas9-mediated gene editing (Fig. 1A).

Since the L1014F, I1011M and I1011V *kdr* mutations have not been reported in IR *Drosophila* field isolates, we first tested whether they conferred resistance to DDT and pyrethroids (permethrin and deltamethrin). The three mutants exhibited differential insecticide resistance profiles: *para*^L1014F^ flies were highly resistant to DDT (Fig. 1B), moderately resistant to permethrin (Supplementary Fig. 2A), but were susceptible to deltamethrin (Supplementary Fig. 2B). In contrast, *para*^I1011M^ flies displayed moderate resistance to DDT and permethrin (Fig. 1B, Supplementary Fig. 2A), while *para*^I1011V^ mutants remained susceptible to all three insecticides even at low concentrations (Fig. 1B, Supplementary Fig. 2A, Supplementary Fig. 2B). Recent molecular modeling studies of the Na^+^ ion channel domain of the VGSC and the conformational consequences of prevalent *kdr* mutations (L1014F, L1014H and L1014S) indicate that substitution of different amino acids even at the same position can have different impacts on DDT binding^11^. Similar alternative conformational effects may explain the observed differential resistance profiles for the *para*^L1014F^, *para*^I1011M^, and *para*^I1011V^ alleles we observed.

### Different *kdr* alleles are associated with distinct fitness costs

We reasoned that the absence of *para*^L1014F^ and *para*^I1011M^ mutations in wild *Drosophila* populations could be due to associated fitness costs. We tested this hypothesis by measuring the effect of these mutations on various fitness parameters. In the absence of insecticide exposure, both *para*^L1014F^ and *para*^I1011M^ significantly reduced lifespan in males (Fig. 1C), but had no detectable impact on embryonic viability (Fig. 1D). Because strain-specific sensitivities to DDT can alter oxidative stress responses in flies^12^, we tested whether *para*^L1014F^ or *para*^I1011M^ mutations impacted resistance to oxidative stress induced by the herbicide paraquat^13,14^. In these experiments, *para*^L1014F^ mutants exhibited significantly elevated resistance to paraquat compared to WT flies, while *para*^I1011M^ mutants remained susceptible (Fig. 1E), revealing again distinct functional outcomes of these *vgsc* mutations. Previous observations also linked mutations in *para* with temperature sensitivity, wherein mutant flies display paralysis at higher temperatures due to altered neuronal activity^15^. Furthermore, a subset of these temperature-sensitive mutants also exhibited resistance to insecticides^16^. Given the ubiquity of the IR L1014F allele in other insects, we hypothesized that temperature sensitivity may contribute to the absence of this particular *kdr* mutation in wild *Drosophila* populations, despite the robust resistance it confers to DDT (Fig. 1B) and permethrin (Supplementary Fig. 2A) exposure. Indeed, *para*^L1014F^ mutants displayed paralysis at higher temperature, while *para*^I1011M^ male mutants were surprisingly more resistant than wild-type (Fig. 1F).

In humans, mutations in VGSC are linked to altered sleep and seizure disorders^17^. Fly models for these disease-causing mutations also manifest significant sleep defects, altered neuronal activity, and neurodegeneration^18–20^. As for the phenotypes discussed above, differential effects on sleep profiles were observed for the various IR alleles we generated. While *para*^L1014F^ mutants followed normal daytime sleep patterns (cumulative and temporal profiles), their night-time sleep was significantly elevated. In contrast, *para*^I1011M^ mutants displayed delayed day-time sleep and elevated night-time sleep (Fig. 1G, H, Supplementary Fig. 3). These distinct physiological phenotypes of the two IR resistant mutations may arise from their differential impact on channel activity^21^ and consequent neuronal functions. Further studies will be required to ascertain the mechanistic basis for these alternative behavioral profiles.

### Development of an allelic drive system to replace the *kdr* 1014 F allele

**Fig. 2:**
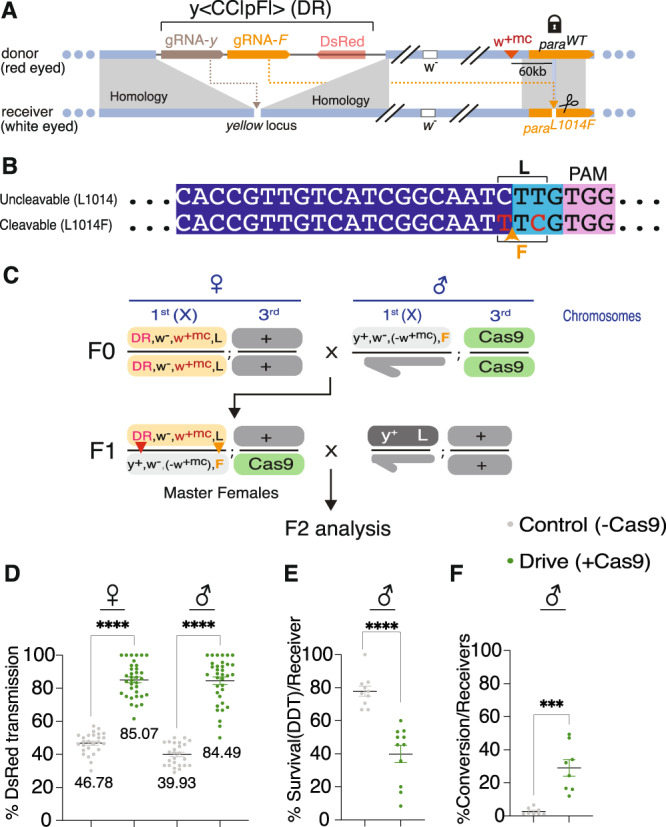
Conversion of the insecticide resistant *para*^L1014F^ allele to *para*^WT^. **A** *y*^<CC|pF|>^ allelic-drive element carrying a DsRed marker and two gRNAs: (1) gRNA-*y* (brown), which sustains copying of the drive element; and (2) gRNA*-F* (orange), which targets the insecticide resistant, gRNA-sensitive *para*^L1014F^ allele (scissors icon) to drive super-Mendelian inheritance of the insecticide-susceptible, uncleavable *para*^WT^ allele (lock icon) when Cas9 is provided in trans. In addition, a *mini-white* marker (*w*^+mc^*, red triangle*), about 0.5 cM from *para*^L1014^ is used to track the donor chromosome carrying the *para*^WT^ allele. **B** gRNA sequence targeting *para*^L1014F^ and its cut-sensitive TTC codon (coding for phenylalanine = F), and the uncleavable CTT *para*^WT^ allele (coding for Leucine = L, marked in red). The cut site is indicated with an arrowhead. **C** Crossing-scheme used to generate F1 “master females”. The yellow shaded X donor chromosome carries the DsRed-marked drive element (*y*^<CC|pF|>^) and the *para*^WT^ allele (labeled L) associated with a *white*^+^ insertion (+*w*^+mc^), which dominantly confers red eye color in the *white*^−^ mutant background. The receiver *white*^−^ X chromosome, which carries the cut-sensitive *para*^L1014F^ allele, but lacks the *w*^+mc^ insertion (−*w*^+mc^ in **C**), is followed through the white^−^ eye color phenotype. A GFP-marked transgene expressing Cas9 (*vasaCas9*) on the third chromosome is depicted in green and wild-type (+) chromosomes in light gray. Red and orange arrowheads indicate copying of the drive element and the *para*^WT^ allele, respectively. **D** Percent transmission of the drive in the presence (DR, green circles) or absence (gray circles) of Cas9 in females and males. Values indicate mean (±s.e.m) percent transmission for each genotype. Data analyzed using one-way ANOVA followed by Sidak’s multiple comparison tests. Chi-square test for DsRed proportions was also performed in presence and absence of Cas9. *p* < 0.0001 was seen in both females and males. **E** Percent survival to 50 ppm DDT in male receiver populations in the presence (green circles) or absence (gray circles) of Cas9. **F** Percentage conversion at 1014 F locus for receiver chromosomes (selected for *w*^−^ eye phenotype) was determined based on the proportion of individual F2 male progeny that carried one or the other allele by sequencing of PCR amplified DNA. These F2 males were collected from F1 master females (*y*^<CC|pF|>^ drive, *w*^+mc^, *para*^WT^/*para*^L1014F^; Cas9/+ ♀ X *w*^−^ ♂). **E**, **F** Means ± s.e.m are plotted. Data were analyzed by Mann–Whitney test^.^ *p < 0.033, **p < 0.0021, ***p < 0.0002, ****p < 0.0001, ns not significant.

We recently developed an efficient CRISPR-based allelic-drive strategy in *Drosophila*^22^ and here, we adapt this system to revert an IR mutation back to the insecticide-susceptible wild-type allele. Because the widespread *para*^L1014F^ allele conferred the greatest resistance to DDT and permethrin, we focused on this mutation for allelic correction. In these experiments, we designed a guide RNA (gRNA-F, Fig. 2B) carried on a gene-drive cassette (*y*^<CC|pF|>^) that selectively targets the *para*^L1014F^ allele for cleavage, leading to its repair from the uncleavable wild-type *para*^L1014^ (=*para*^WT^) allele. We placed gRNA*-*F under control of the ubiquitously active U6 promoter in the *y*^<CC|pF|>^ transgenic drive element, which also expresses an additional gRNA (gRNA*-y*) that targets the insertion site of the *y*^<CC|pF|>^ element for cleavage within the *yellow* (*y*) locus to promote self-copying and super-Mendelian inheritance of that element (Fig. 2A). We followed inheritance of the *y*^<CC|pF|>^ element by the DsRed fluorescent protein marker, expressed in eyes under the control of the *3XP3* promoter.

We first assessed performance of the *y*^<CC|pF|>^ allelic-drive element ±Cas9 in two-generation test crosses (Fig. 2C). We chose a *mini-white* insertion tightly linked (~0.5 cM) with the uncleavable *para*^WT^ donor allele to distinguish it from the cut-sensitive *para*^L1014F^ receiver allele. F2 progeny inheriting the *white*^−^ (*w*^−^) “receiver” chromosome (hereafter referred as F2 *w*^−^ receiver progeny) were either unmodified (*para*^L1014F^) or converted (para^WT^) (Fig. 2A, C).

We assayed super-Mendelian transmission of both the *y*^<CC|pF|>^ gene cassette and allelic-drive of the *para*^WT^ allele. As per the cross-scheme depicted in Fig. 2C, a Cas9 expressing transgene (*vasa-Cas9*) was provided from an unlinked third chromosomal source. Introduction of Cas9 led to a substantial increase of *y*^<CC|pF|>^ (DsRed^+^) transmission in F2 progeny, compared to F2 progeny from control (-Cas9) crosses, (46.8% to 85.1% in females and 39.9% to 84.5% in males, Fig. 2D), confirming that the drive element copies with similar efficiency to other drives inserted at this same site^22–25^. The fraction of F2 *w*^−^ receiver progeny, however, was not altered significantly in presence of the Cas9 source (~50% for both control and drive) (Supplementary Fig. 4), indicating that DNA cleavage directed by gRNA-F did not result in frequent production of lethal mutations. Next, we tested F2 *w*^−^ adult receiver progeny for insecticide resistance. Such receiver progeny derived from *y*^<CC|pF|>^; Cas9 bearing mothers displayed a significantly lower percent survival in presence of DDT (39.9% in males, 62% in females) compared to F2 *w*^−^ receiver flies from control crosses (75.5% in males, Fig. 2E, 85.18% in females, Supplementary Fig. 5B). These results demonstrate biased inheritance of the uncleavable *para*^WT^ allele mediated by cleavage and conversion of the IR *para*^L1014F^ allele to *para*^WT^ allele. Cas9-dependent conversion from F- > L at the 1014 site was confirmed by genotyping individual F2 males. Among F2 *w*^−^ receiver males from *y*^<CC|pF|>^ control mothers, the proportion of L1014 wild-type allele was very low (2.7%), consistent with the low recombination rate expected from the close association (~60 kb) between the *para* locus and the *w*^+mc^ insertion. In contrast, F2 *w*^−^ receiver males derived from *y*^<CC|pF|>^; Cas9 mothers exhibited a >10-fold increase (29.0%) in the proportion of the uncleavable, wild-type L1014 allele (Fig. 2F, Supplementary Fig. 5D). A similar trend was observed in F2 *w*^−^ receiver females, where we observed a modest but significant decrease in the percentage of the 1014F allele (Supplementary Fig. 5A, C). However, because females have two X chromosomes, it was difficult to assess the precise conversion frequencies, which are more accurately determined in males. Cumulatively, these results demonstrate a ~30% frequency of allelic conversion from 1014F to L1014, resulting from Cas9-dependent allelic-drive induced by the *y*^<CC|pF|>^ drive element.

### The *kdr* 1014 F allelic-drive reverses insecticide resistance in population cages

Given the efficient super-Mendelian inheritance of both the *y*^<CC|pF|>^ drive element and *para*^WT^ allele in two-generation test crosses, we next examined their performance over multiple generations in population cages. We seeded three replicate cages with equal numbers of males and females. Half of the females (25% of total flies) were heterozygous for the *y*^<CC|pF|>^ allelic-drive, Cas9, and L1014 *para*^WT^ allele (comprising 16.7% of all *para* alleles) and the other half of the females (25% of total flies) as well as all males (50% of total flies) carried the IR 1014 F allele. One half of the F1 progeny were monitored for DsRed and DDT resistance phenotypes as well as for genotypes (L1014 versus 1014F sequences) at each generation, while the other half of that population was used to seed the next generation (without sorting for gender).

**Fig. 3:**
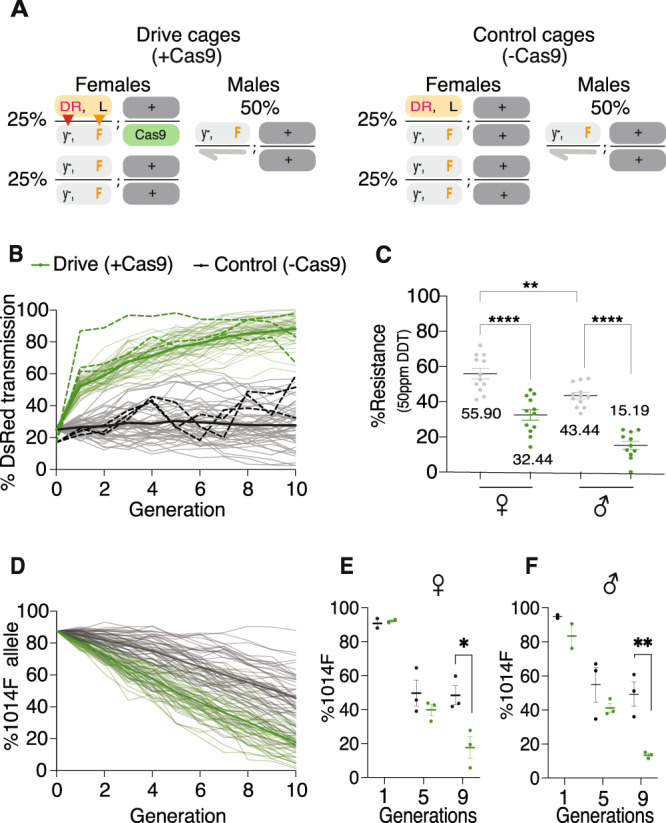
Dynamics of 1014 F conversion over 10 generations in caged populations. **A** Virgin ‘master females’ carrying the *y*^<CC|pF|>^ allelic-drive (DR) and Cas9 were seeded among receiver *para*^L1014F^ flies at a ratio of 25%:75% (*n* = 100; *N* = 3). **B** Model-predicted dynamics of the *y*^<CC|pF|>^ drive element. Mathematical simulations were run using fitted drive parameters and estimated fitness costs. 50 stochastic predictions are plotted in thin lines and their mean in thick lines. Experimental data from 3 independent replicates was overlayed as dotted lines. **C** Resistance to 50 ppm DDT was tested separately for generations 9–12 (Fig. S6E, F) and averaged. Percent resistance to DDT exposure (assayed by survival) in the presence (drive) or absence (control) of Cas9 combined for all 4 generations is plotted and analyzed using one-way ANOVA followed by Sidak’s multiple comparison tests. **D** Dynamics of 1014F allelic frequency was modeled separately from the *y*^<CC|pF|>^ element. The model predicted a decrease in 1014F levels for both control and drive populations with faster decline in presence of Cas9. **E**, **F** The proportion of the 1014F allele was measured by deep sequencing of females and males at generation 1, 5 and 9. The percentage of 1014F alleles in the presence (green dots) or absence (gray dots) of Cas9 was plotted and analyzed using multiple unpaired *t* tests. Data plotted as mean ± s.e.m. *p < 0.033, **p < 0.0021, ***p < 0.0002, ****p < 0.0001, ns not significant.

Cage experiments were conducted in *y*^−^ *w*^−^ genetic backgrounds to preclude known mating and fitness advantages associated with wild-type *y*^+^ and *w*^+^ alleles^26,27^. Drive cages included the *y*^<CC|pF|>^ element on the X and *vasaCas9-GFP* on the 3rd chromosome, while control cages lacked the Cas9 source (Fig. 3A). This scheme permitted assessment of the relative fitness costs associated with different alleles (for example the *y*^<CC|pF|>^ element versus a *y*^−^ point mutation, or the *para*^1014L^ versus *para*^1014F^ alleles) in the presence or absence of Cas9, as well as evaluating Cas9-dependent drive performance. The fitness costs calculated from the control (–Cas9) cages were used for predictive modeling of population dynamics for the *y*^<CC|pF|>^ drive (Fig. 3B, Supplementary Fig. 6C) and 1014F allele (Fig. 3D, Supplementary Fig. 6D) and were found to be consistent with two of the three observed cage replicates. The initial divergence of one cage from the others, and hence the averaging model, could stem from variation in certain fitness costs or reflect statistical variation due to sampling, however, this discrepancy disappeared by generation 10. Thus, the *y*^<CC|pF|>^ element achieved ~80% introduction (final frequency) in all +Cas9 drive cages while increasing only modestly in control (−Cas9) cages (Fig. 3C). In such experiments, generation of uncleavable mutations by non-homologous end joining (NHEJ) events at the *yellow* locus may limit the spread of the *y*^<CC|pF|>^ element as previously reported^22–24^. The frequency of the unlinked Cas9 (GFP+) source remained stable over 10 generations suggesting that the *vasaCas9* transgene did not impose a detectable fitness cost, even when associated with the drive element (Supplementary Fig. 6A), a phenomenon that has been observed in other contexts^28,29^. The total population size and sex ratio also remained stable for both the drive and control cages throughout the experiments, suggesting that no significant fitness-induced distortions were operative (Supplementary Fig. 6B).

Regarding the primary objective of these experiments, we also evaluated the DDT resistance status of drive (+Cas9) and control (−Cas9) cage populations over time. Based on prior studies in which equilibrium final frequencies between split-drive cassettes and NHEJ alleles were attained by generations 8/9^28^, we tested generations 9–12 for DDT resistance. We observed a dramatic reversal of DDT resistance from the initial introduced percentage of 83.3% to 15.2 % in males of the drive containing cages (+Cas9) and a more modest, but significant, reduction in females (32%). Control (−Cas9) cages displayed less dramatic reductions in IR (43.3% for males; 55.9% for females), which presumably reflects negative selection based on the fitness costs associated with the 1014F allele (Fig. 3C, Supplementary Fig. 6E, Supplementary Fig. 6F, Supplementary Table 5, Supplementary Table 6). This IR reversal was further confirmed by sequence analysis of males and females from drive and control populations at the 1014 site at three sampled generations (generations 1, 5, and 9). At generation 5, we observed modest parallel decrements in 1014F allele in both +Cas9 drive and −Cas9 controls. However, by generation 9, the frequency of the 1014F allele was greatly reduced in both male and female drive populations. Among males, 1014F representation had dropped precipitously to 13.4% in the drive cages, while decreasing only to 49.3% in controls (Fig. 3F). Similarly, among females, the 1014F frequencies fell to 17.5% in the drive populations compared to 48.3% in control populations (Fig. 3E, Supplementary Fig. 7). Although IR allelic frequencies were not significantly different between drive males and females (i.e., 13.4% versus 17.5%, respectively), we observed a notable difference in their percent survival when exposed to 50 ppm DDT (15.2% versus 32.54%, respectively - Fig. 3C). This greater residual survival observed in females most likely reflects the presence of *para*^WT^*/para*^L1014F^ heterozygotes, which display intermediate IR levels (Supplementary Fig. 8). Additionally, physiological differences between males and females may contribute to their differential resistance to DDT^30^.

In these experiments, recovery of NHEJ events at the *para* 1014 site was exceedingly rare (Fig. S5A, D). One possible explanation is that most NHEJ mutations that may have been created at the conserved gRNA target site are likely to be non-functional, resulting in lethal mosaicism in females^22^, and lethality in males. However, as mentioned above, we did not find a significantly biased reduction in the proportion of F2 *w*^*−*^ receiver progeny (Supplementary Fig. 4), suggesting that such mutant lethal alleles are not likely to be created at an appreciable rate. Our results from cage trial experiments show that 90% introgression of the L1014 wild-type allele is achieved at generation 9, which is delayed compared to the rapid spread of the drive element (~90% introgression at generation 3). This kinetic difference suggests that cleavage induced by gRNA-F may be less efficient than for gRNA-*y*, as inferred by estimates from model fitting (Supplementary Tables 5 and 6). However, because gRNA-F generates few NHEJ alleles, its modest, but clean, cleavage profile supports a delayed but eventually superior drive outcome. Another factor likely to contribute to the extensive reversal in IR obtained in these drive experiments is the synergistic action of two independent selective processes^24^ wherein the allelic drive promoting Super-Mendelian inheritance of the L1014 allele (e.g., mean copying efficiency to receiver chromosomes = 0.29) acts in combination with the greater relative fitness of the wild-type L1014 versus 1014F allele (e.g., median fitness cost of 1014F allele in males = 0.28) (Fig. 1C–F, Supplementary Tables 5 and 6), a feature also likely to be relevant in field contexts^5,7,9,31–34^. We note that this synergy between the independently acting gene-drive and positive selection for the 1014 allele in the driving configuration (+Cas9) also may be reflected by the reduced variance in allelic frequencies between cages observed in generation 9 (Fig. 3F, most obvious in males) compared to the action of positive selection alone (−Cas9), a phenomenon we have observed in other studies^24^. Presumably, the likelihood of 1014 L allele inheritance is increased as a result of these two cooperative processes.

## Discussion

We (this study) and others^35^ have exploited the powerful genetic tools available in *Drosophila* to establish models for prevalent insecticide-resistance mutations in VGSC. Here, we report differential physiological effects and responses to stresses for three different *kdr* mutations, and most importantly, provide the first proof-of principle for allelic-drive mediated reversal of IR conferred by *para*^1014F^ to the native *para*^L1014^ insecticide susceptible state, in the absence of insecticides. In more real-world conditions applied to disease vectors such as mosquitoes transmitting malarial parasites or crop pests, one anticipates uninterrupted insecticide use. In such situations where local control of insecticide use is possible (e.g., farmers treating their own fields) alternating between two different categories of insecticides impinging on separate targets (e.g., pyrethroids versus organophosphates or carbamates targeting Acetylcholinesterase), coupled with allelic-drive reducing IR to the fallowed insecticide, could provide a sustainable approach to achieving local reductions in IR frequency. Another synergistic strategy would be to neutralize mechanisms of metabolic resistance. Metabolic IR targets (often P450-related genes) are more challenging targets as they are less readily identified than target site mutations and comprise a complex set of potential mechanisms including gene duplications, transposon insertions, other types of chromosomal rearrangements, and alterations of cis-regulatory sequences^36–39^. Nonetheless, if identified as individually contributing to overall IR phenotypes, any one of these alterations could be reversed either by targeting specific duplicated alleles for mutagenesis, small regions could be deleted by the copy-grafting form of allelic drive in which a cleavage resistant cut site is associated nearby a favored allelic variant, or two gRNAs could be used to delete and replace larger genome segments including cis-regulatory regions^40^ or gene-cassettes^24^. Such genetic strategies could be integrated with traditional measures and regular monitoring of new IR alleles emergence for an efficient multipronged vector control approach.

Allelic-drive systems such as those discussed above could also be combined with gene drives designed to modify insect populations. For example, drive systems have been developed in mosquitoes^41,42^ and fruit flies^28,29^ that are capable of efficiently disseminating cargo (e.g., anti-malarial effector genes in mosquitoes) in laboratory populations. Such autosomal drives are indeed likely to be yet more effective than the X-linked drive element employed in this proof-of-concept study since they can copy in the germlines of both males and females. For example, gRNAs targeting IR alleles could be added to such drive systems in anopheline mosquitoes resulting in dual population modification to reduce parasite transmission while maintaining efficacy of standard vector control measures. It may also be possible to conduct genetic screens to identify target site variants conferring greater than wild-type susceptibility to an insecticide. Introducing such hyper-sensitive alleles through allelic drives could ultimately allow reduction of insecticide applications required to manage local insect populations. Importantly, genetic methods to reduce the prevalence of IR and to combat vector-borne diseases need to be considered as a new tool to be integrated into existing vector control protocols including use of LLINs, indoor residual spraying, anti-parasite medicines, and sanitation measures. Reducing the incidence of IR via allelic-drive offers potential for synergism with the first two insecticide-dependent measures, which have been credited as the primary factor in reducing malarial burdens over the past two decades^2,43–46^.

Multiple models and studies have established significant decrements in LLINs efficacy due to increasing insecticide resistance. These models also indicate that efficacy of current vector control methods^47,48^ could be preserved if the prevalence of IR was maintained below 30%, which we surpassed (<15% IR) in cage experiments of lab populations. However, the variability in natural populations could impact the efficacy of the drive if a common variant target site allele were present in the target population. Although such insecticide target site allelic variants at the gRNA cut site are not expected to be common given the highly conserved nature of this coding region of the *vgsc* gene and the small number of recent independent emergence of such alleles^49,50^, there are two possible approaches to mitigating such a problem should it arise. First, one could create two different allelic drives with gRNAs targeting the two most frequent allelic 1014 F variants and release them together or sequentially into the target population to achieve the required level of allelic correction. Second, the alternative allelic drive strategy of copy-grafting^22^ could be employed in which one associates a highly conserved cleavage resistant site close to the preferred L1014 insecticide susceptible allele. Such engineering might permit replacement of any *kdr* sequence variants located in the proximity (within ~25 bp) to such a unique engineered site.

Cluster-randomized clinical trials of LLINs confirm the epidemiological benefits of augmenting performance of pyrethroids in bednets^51–54^. In combination with transient inherently self-limiting drives or other systems in which the drives and Cas9 transgenes are inserted into essential loci^23,28,29^, it should be possible to increase the prevalence of naturally occurring alleles facilitating vector control efforts (e.g., wild-type insecticide susceptible *vgsc* alleles or natural variants of the mosquito FREP1 gut protein conferring resistance to malarial parasites^55–57^) without leaving any vestige of the transgenic machinery. We conclude that this flexible new platform will enable study and control of IR mutations ultimately to allow limited and more efficient use of insecticides for effective vector control efforts.

## Methods

### Fly strains

vasaCas9-GFP (BL# 79006), y[1] w[67c23] P{y[+mDint2]w[+mC]= P{EPgy2}CG9902[EY05861], and (BL# 15811) strains were procured from Bloomington Drosophila Stock Centre (BDSC). Other lines used in the study were generated in-house.

### Generation of insecticide resistance mutant flies

The IR flies were generated at the *para* genomic locus using two steps of CRISPR-Cas9 mutagenesis. In the first step, a double stranded break was inserted at L1014 locus using gL1014 (ttcgtattcttcatcatatt). Using homology arms for *vgsc*, 3XP3 > GFP was inserted at the cut site. Transformants were selected as presence of GFP fluorescence in the eye. In the second step, GFP was deleted using two gRNAs (g1: ttcgtattcttcatcatatt, g2: Gtagatgttcgtttcacgaat) and replaced using homologous template containing desired mutations (L1014F/I1011M/I1011V). The transformants were screened with loss of GFP and sequenced for presence of the mutation (Fig. 1A). The males with unsuccessful homology-based repair did not survive as *para* is an X-linked essential gene. This male lethality was used as additional criteria for screening.

### Construction of *y*^<CC|pF|>^ element and transgenic flies

The allelic drive element *y*^<CC|pF|>^ was constructed from the *y*^<CC|N|>^ element described in Guichard et al, which contains two gRNAs, one targeting *yellow* for self-propagation (gRNA-*y*) and one targeting the *Notch* locus (gRNA-*N*) for allelic drive, plus the DsRed fluorescent marker expressed under the control of the eye-specific 3xP3 promoter. gRNA-*N* was replaced with gRNA-*F*, which targets the IR L1014F allele of *para* (gRNA-F: gcgttgtcatcggcaatttcg). DNA encoding gRNA-*F* plus two homology arms matching sequences directly flanking gRNA-*N* (20 nt on each side) was synthesized by IDT (gBlocks). Replacement of the gRNA sequence was obtained through Gibson assembly using the NEB kit #E5510S. The full sequence of the assembled plasmid was verified and is provided in Supplementary Note 2. The *y*^<CC|pF|>^ construct was then injected in *w*^−^ stock by Rainbow transgenics. Positive *y*^<CC|pF|>^ transformants were screened first through DsRed fluorescence in the eyes, followed by sequencing of the inserted cassette after PCR amplification of genomic sequences.

### Insecticide resistance assays

Different concentrations of insecticides used in the assay were prepared in acetone. For each repeat experiment, all solutions were prepared fresh from the received powders, and working solutions were formulated as 1 ppm = 1 mg/L of the insecticide powder. For example, in Figs. 2 and 3, 500 μl of 50 ppm (50 mg/ml) of DDT solution = 25 mg of DDT was used per vial per experiment as has been previously described^16^^,^^58^. Glass scintillation vials were coated with 500 μl of desired concentration of given insecticide using an automated rotator to evaporate all the acetone have uniformly coated vials. These vials were used the next day^58^. 25–30 flies per vial were transferred by tapping and the vials were closed with cotton plugs soaked in 5% sucrose solution. Care was taken not to expose flies to CO_2_ on the day of the assay. Dead flies were recorded 24 h post exposure. For Fig. 1B, Supplementary Fig. 2A, Supplementary Fig. 2B, male flies were used. For Figs. 2E and 3C, assayed male and female flies were collected and processed separately for genotyping.

### Embryo hatchability

2–4-day-old females were mated with males of same genotype. 100–120 embryos from the resulting cross were collected at 24 h for each genotype and lethality (non-hatched embryos) was measured at 48 h.

### Lifespan

Protocol adopted from Piper and Partridge^59^. Briefly, 1–3 old males were collected for each genotype and distributed in 12–15flies/vial, at least 10 vials per genotype. The deaths were recorded every alternate day and flies were transferred to fresh food twice a week. The assay was done at 25 °C.

### Oxidative stress assay

Standard fly food with 20 mM paraquat added was prepared. 2 ml of food was used per vial. Twenty-five 3-5-day-old female flies of each genotype were distributed per vial. Five independent replicates were done. Flies were maintained at 25 °C. Deaths were recorded 24 h and 48 h post exposure.

### Temperature sensitivity assay

3–5 days males were distributed as 20 flies per empty vial with wet cotton plug. Experiment was repeated in four independent replicates. Flies were exposed to 37 °C for 4 h. The vials were tapped, and the movement of flies was recorded for 1 min. A control set at 25 °C was monitored with no significant difference in mobility.

### Sleep behavior assay

All flies were raised on standard molasses-yeast cornmeal vials at 25 °C. To measure sleep, we used 4–7 days old female and male flies. Individual flies were loaded into glass tubes containing 5% sucrose and 1% agarose as the food source at one end and monitored by the *Drosophila* Activity Monitoring System (TriKinetics). Flies were entrained for 2 days under 12 h:12 h light:dark cycles before activity data were collected for analysis. Sleep is identified as periods of inactivity for at least 5 min^60^. Sleep assays were performed under full-spectrum lights (Waveform Lighting) during the light period at 25 °C.

### Active genetic safety measures

All experiments were done using split-drive system. In line with UCSD Biosafety Committee-approved protocol, split drive experiments are performed in an ACL1 insectary in plastic vials that are autoclaved prior to being discarded.

### Sample preparation for sequencing

Briefly, 30–50 male or female flies per replicate were crushed in 500 μl homogenization medium (0.1 M Tris, 0.1 M EDTA,1%SDS, 0.5% diethylpyrocarbonate) and incubated at 65 °C for 30 min. The DNA was precipitated using 100 μl of 8 M potassium acetate and Isopropanol^61^. The extracted gDNA is amplified using *vgsc* specific primers followed by adapter primers and run on Illumina NOVOseq platform. Data were analyzed using CRISPResso2 and CrispRVariants pipelines.

*vgsc* forward: 5′ACACTCTTTCCCTACACGACGCTCTTCCGATCTagcttcatgatcgtgttcc 3′

*vgsc* reverse: 5′GACTGGAGTTCAGACGTGTGCTCTTCCGATCTgccatggttagaggcgataagtc 3′.

### Modeling analysis

Model fitting was carried out using a discrete-generation adaptation of the Mosquito Gene Drive Explorer (MGDrivE)^62^. A likelihood-based Markov chain Monte Carlo (MCMC) procedure was used to estimate gene drive efficacy and genotype-specific fitness costs, employing initial parameter projections from the single-pair test crosses providing Maximum a Posteriori estimates and 95% credible intervals for each parameter^63^. Mendelian inheritance was assumed except under co-occurrence of the Cas9 and gRNA constructs when the split-drive design allowed active cleavage of the target chromosome and the possibility of super-Mendelian inheritance. In females, when cleavage occurred, a fraction of the cut alleles could be properly repaired via HDR, and the remaining cut alleles underwent NHEJ repair, generating in or out-of-frame resistant alleles, at both *yellow* and *para* loci. At *yellow*, HDR results in drive, and super-Mendelian inheritance, of the gRNA construct. At *para*, HDR possibly results in allelic conversion, depending on the sister chromatid. In males, which are monoploid for the X-chromosome, there is no wild-type *yellow* locus available for cleavage when the gRNA construct is present. Additionally, there is no repair template for the *para* locus, thus repair involves direct ligation or resection and generation of NHEJ alleles. Fitness costs were implemented as copy-number dependent reductions in female fecundity or male mating competitiveness in the presence of the 1014F allele at the *para* locus. Additionally, as *para* is a haplosufficient essential gene, females with two out-of-frame NHEJ alleles (or males with one) were considered unviable. More details on the model implementation and likelihood function used in the model fitting can be found in the Supplementary note 1. The results of each fit, using the estimated parameters, are plotted in Fig. 3B, D along with corresponding experimental cage trial data. Parameter descriptions and estimates for each set of cage trials are provided in Supplementary Tables 5 and 6.

Simulated model trajectories for Fig. 3 were generated using a stochastic implementation of the discrete-generation model. At each generation, adult females mate with males, thereby obtaining a composite mated genotype (their own, and that of their mate) with mate choice following a multinomial distribution determined by adult male genotype frequencies, modified by mating efficacy. Egg production by mated adult females then follows a Poisson distribution, proportional to the genotype-specific lifetime fecundity of the adult female. Offspring genotype follows a multinomial distribution informed by the composite mated female genotype and the inheritance pattern of the split allelic-drive system. Sex distribution of offspring follows a binomial distribution, assuming equal probability for each sex. Female and male adults from each generation are then sampled equally to seed the next generation, with sample size proportional to the average size of the cage trials at that generation, following a multivariate hypergeometric distribution. All simulations were performed and analyzed in R^64^. Code is uploaded on zenodo at DOI: 10.5281/zenodo.5715691.

### Ethical conduct of research

We have complied with all relevant ethical regulations for animal testing and research and conformed to the UCSD institutionally approved biological use authorization protocol (BUA #311).

### Statistics and reproducibility

All data shown here are from at least three independent experiments. No data were excluded from the analysis. The sample size was decided based on earlier similar experiments and consistency of the data during experiments. No blinding was required as experiments were done by same investigator.

Data were analyzed and plotted using GraphPad Prism 9.2.0. Mean ± s.e.m. was plotted unless otherwise indicated. For comparison between three or more samples, one-way ANOVA and Sidak *post hoc* test was used. For more than one group’s experiments, two-way ANOVA was used. The significance was considered as *p* < 0.05 (*), *p* < 0.01 (**), *p* < 0.001 (***), or *p* < 0.0001 (****).

## Supplementary information

Supplementary Information

## Data Availability

The sequencing data is uploaded to NCBI and available online. The accession numbers are PRJNA757741 for F2 female deep sequencing data, Supplementary Fig. 5A; PRJNA758048 for F2 male deep sequencing data, Supplementary Fig. 5D; PRJNA758082 for cage trails deep seq data (gen 1 5 and 9), Supplementary Fig. 7. The modeling data are uploaded at 10.5281/zenodo.5715691. Source data are provided with this paper.

## Source data

Source Data
