# Supplementary material for: Reversing insecticide resistance with allelic-drive in Drosophila melanogaster
Source: Nat Commun. 2022 Jan 12;13:291. doi: 10.1038/s41467-021-27654-1 (PMC8755802; doi:10.1038/s41467-021-27654-1)
Supplement: Supplementary file 1 — Supplementary Information [file 41467_2021_27654_MOESM1_ESM.pdf]

Supplementary Materials for

Reversing insecticide resistance with allelic-drive in *Drosophila melanogaster*

Bhagyashree Kaduskar[1,2 ,3‡], Raja Babu Singh Kushwah[1,2 ,3‡], Ankush Auradkar[2], Annabel Guichard[2,3], Menglin Li[4,5], Jared B. Bennett[6], Alison Henrique Ferreira Julio[7], John M. Marshall[8,9], Craig Montell[4,5], Ethan Bier[2,3*]

Corresponding author email: ebier@ucsd.edu.in

This PDF file contains:
Supplementary figures 1-10
Supplementary table 1-6
Supplementary note 1
Supplementary note 2

Supplementary Figure 1

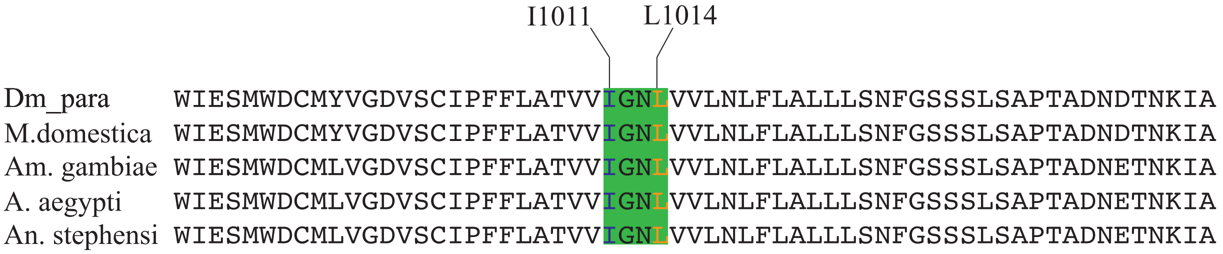

Supplementary Figure 1: Different insects across genus and species show 100% identity near the insecticide target site. The kdr mutations used in these studies are at site I1011(blue, I1011M/V) and L1014(orange, L1014F)

Supplementary Figure 2

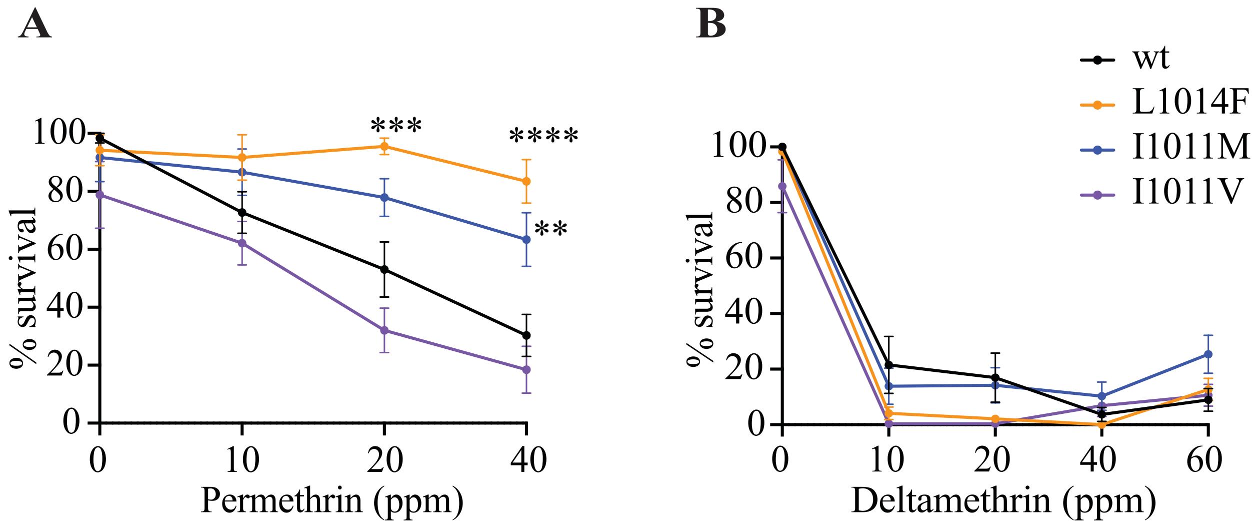

Supplementary Figure 2: Survival plots for Permethrin and Deltamethrin at different concentrations, exposed for 24h. Mean ± s.e.m is plotted. Data was analyzed by 2-Way ANOVA with Sidak multiple comparisons test.

* = p < 0.033, ** = p < 0.0021, *** =p < 0.0002, **** = p<0.0001, ns = not significant.

Supplementary Figure 3

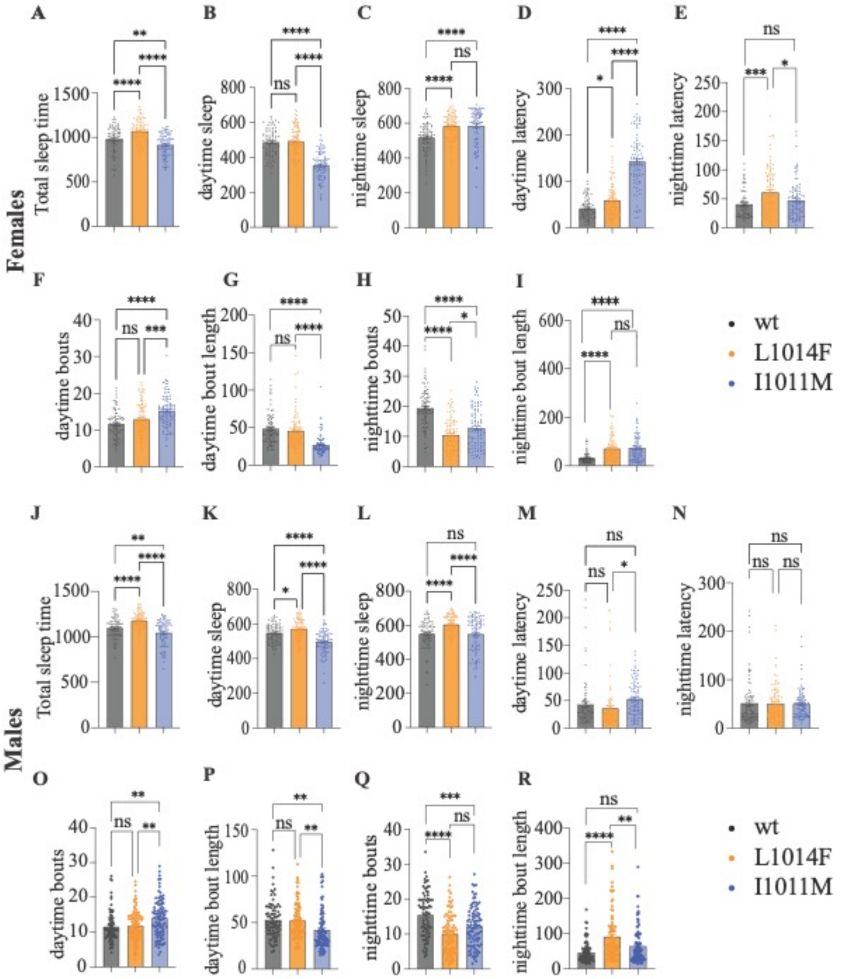

Supplementary Figure 3: **Altered Sleep parameters in *L1014F* and *I1011M* flies.** (A-I, J-R) Total sleep time, daytime sleep and nighttime sleep of *para*^{WT} (dark grey), *para*^{L1014F} (orange) and *para*^{I1011M}(blue) females or males housed under 12hr:12hr light:dark cycle. Sleep is calculated from the number of minutes flies were asleep during ZT0-ZT24(A), ZT0-ZT12 lights on (B) or ZT12-ZT24 lights off (C). (D and M) Daytime sleep latency of *para*^{WT}, *para*^{L1014F} and *para*^{I1011M} females or males. Daytime sleep latency is calculated from time after lights on to first sleep bout. (E and N) Nighttime sleep latency of *para*^{WT}, *para*^{L1014F} and *para*^{I1011M} females or males. Nighttime sleep latency is calculated from time after lights off to first sleep bout. (F, H, O and Q) Average daytime or nighttime sleep bouts per day of *para*^{WT}, *para*^{L1014F} and *para*^{I1011M} females or males. (G, I, P and R) Average daytime or nighttime sleep bout length of *para*^{WT}, *para*^{L1014F} and *para*^{I1011M} females or males. n =75-96 flies per genotype from 3 biological replicates. All data are averaged over the span of 96h. Mean ± SEM is shown. * = $p < 0.05$, ** = $p < 0.01$, *** =$p < 0.001$, **** = $p<0.0001$, ns = not significant.

Supplementary Figure 4

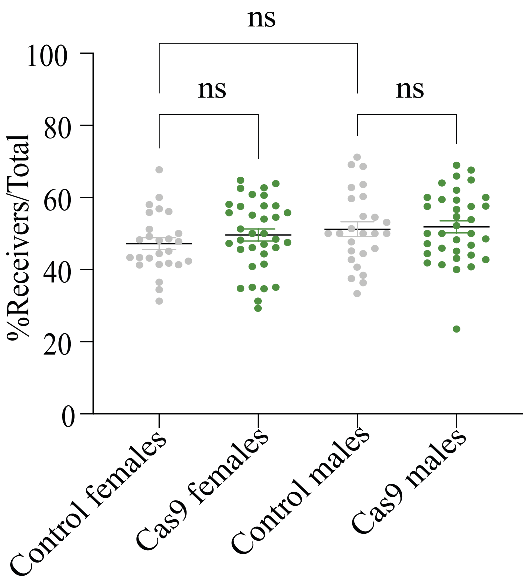

Supplementary Figure 4: The w-F2 males and females from Cross scheme in Fig2C are counted and plotted as percentage of the total population. The receivers are about 50% of the total population, consistent with Mendelian ratios both for Control (grey circles) and Drive (green circles) populations. This indicates no significant costs in egg-to-adult viability of Cas9 transgene or any lethal mosaicism. Mean ± s.e.m with scattered plot is plotted. Data was analyzed by 2-Way ANOVA with Sidak multiple comparisons test. Ns= not significant

Supplementary Figure 5

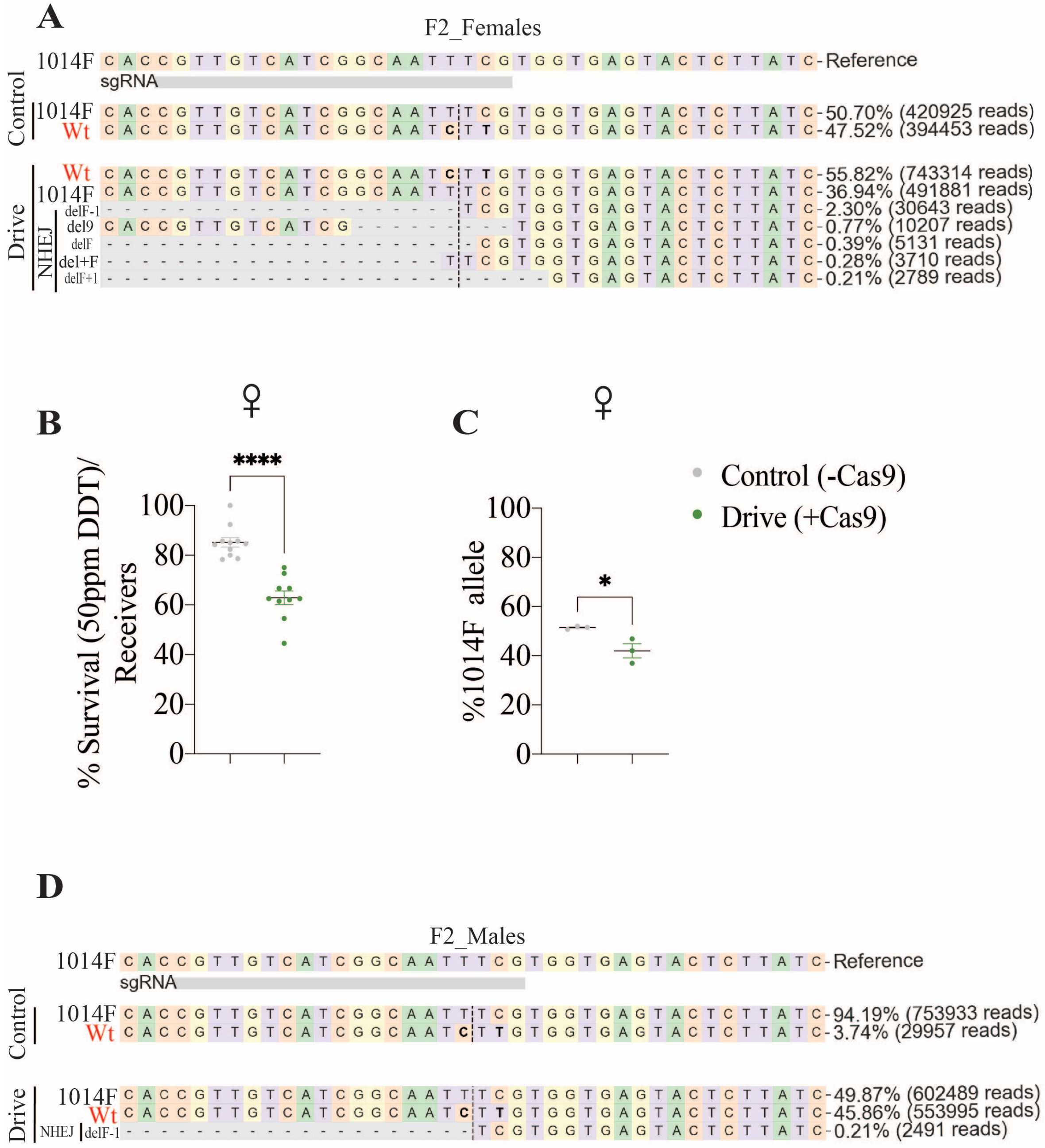

Supplementary Figure 5: (**A**) Representative data from Amplicon sequencing of w- receiver F2 progeny from control and master females as shown in cross scheme in Fig2C. In controls, there is ~50% of each allele. In Drive (+Cas9) progeny, the 1014F proportion is reduced to ~37% with ~7% NHEJ events. Females have 2 X chromosomes and the NHEJ events captured in bulk sequencing could be somatic. **(B)** Resistance to 50ppm DDT was tested for w-F2 females. As compared to controls (85.18%), the Cas9 containing flies showed significantly lower resistance (62%). **(C)** Allelic frequency calculated from amplicon sequencing data suggest a small but significant decrease in 1014F allele percentage from 51.4% in controls to 42% in Drive (+Cas9) progeny. Mean ± s.e.m is plotted. Data was analyzed Mann-Whitley test. * = p<0.05, **** = p<0.0001 **(D)** Representative amplicon sequencing data for w-F2 males.

Supplementary Figure 6

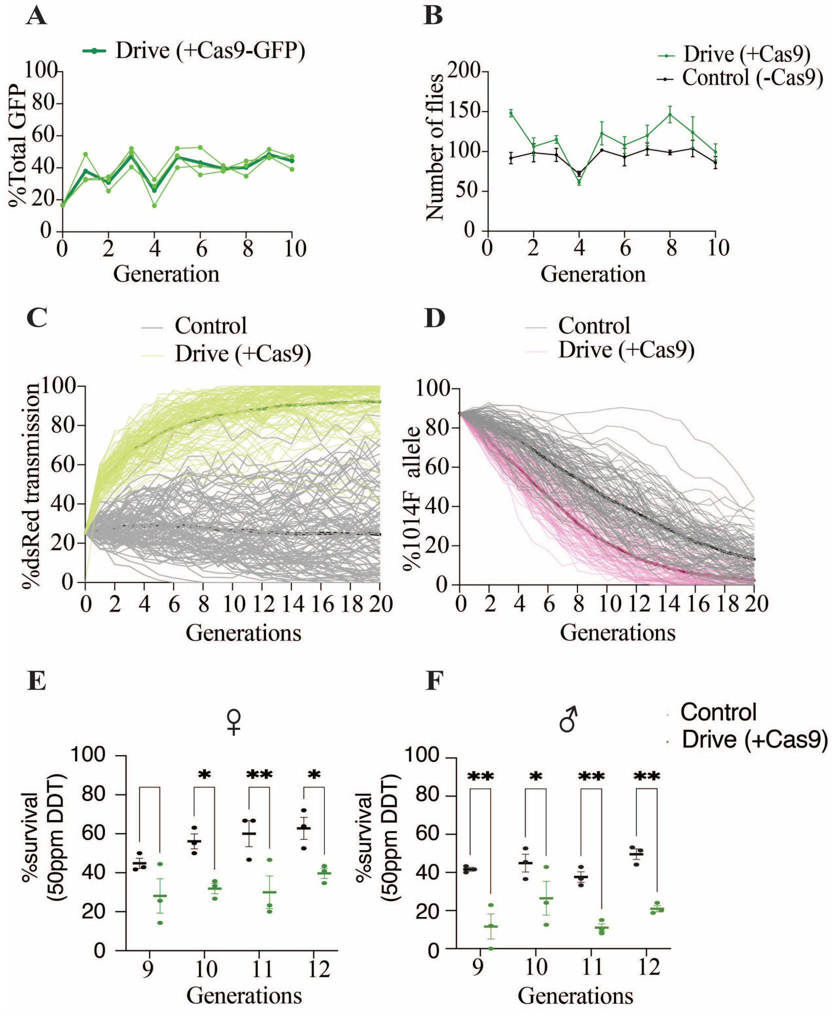

Supplementary Figure 6: (A) vasaCas9-GFP proportions. The thin lines represent 3 independent cages while thick line represent the mean values. and **(B)** total populations remain stable across generations in cage trial experiments set according to schematics in Figure 3a. Mean ± s.e.m is plotted. **(C, D)** Mathematical modeling extended through 20 generations estimating the terminal percentages of the DsRed marked drive cassette **(C)** inserted at the yellow locus (±Cas9), and **(D)** the prevalence of the 1014F allele (versus the wild-type allele = 100% - 1014F percentage). Note that the prevalence of the drive casette is predicted to reach an equilibrium frequency with drive-resistant alleles (due to NHEJ alleles being generated at the *yellow* gRNA cut site), while the 1014F allele is predicted to become fully replaced by the wild-type allele (due to the drive + fitness advantage of wild-type allele over the 1014F allele and the virtual lack of NHEJ generation by the vgsc 1014F targeting gRNA. **(E, F)** %Survival at 50ppm DDT was plotted for control (grey dots) and Drive+ (green dots) for generation 9 to 12. The values across generation for control or Drive are comparable showing no significant fitness cost. The %survival differ significantly between control and Drive at gen 10 to 12 for females **(E)** and generation 9 to 12 for males **(F)** Mean± s.e.m is plotted. Data analysed using 2-WAY ANOVA followed by Sidak multiple comparison.

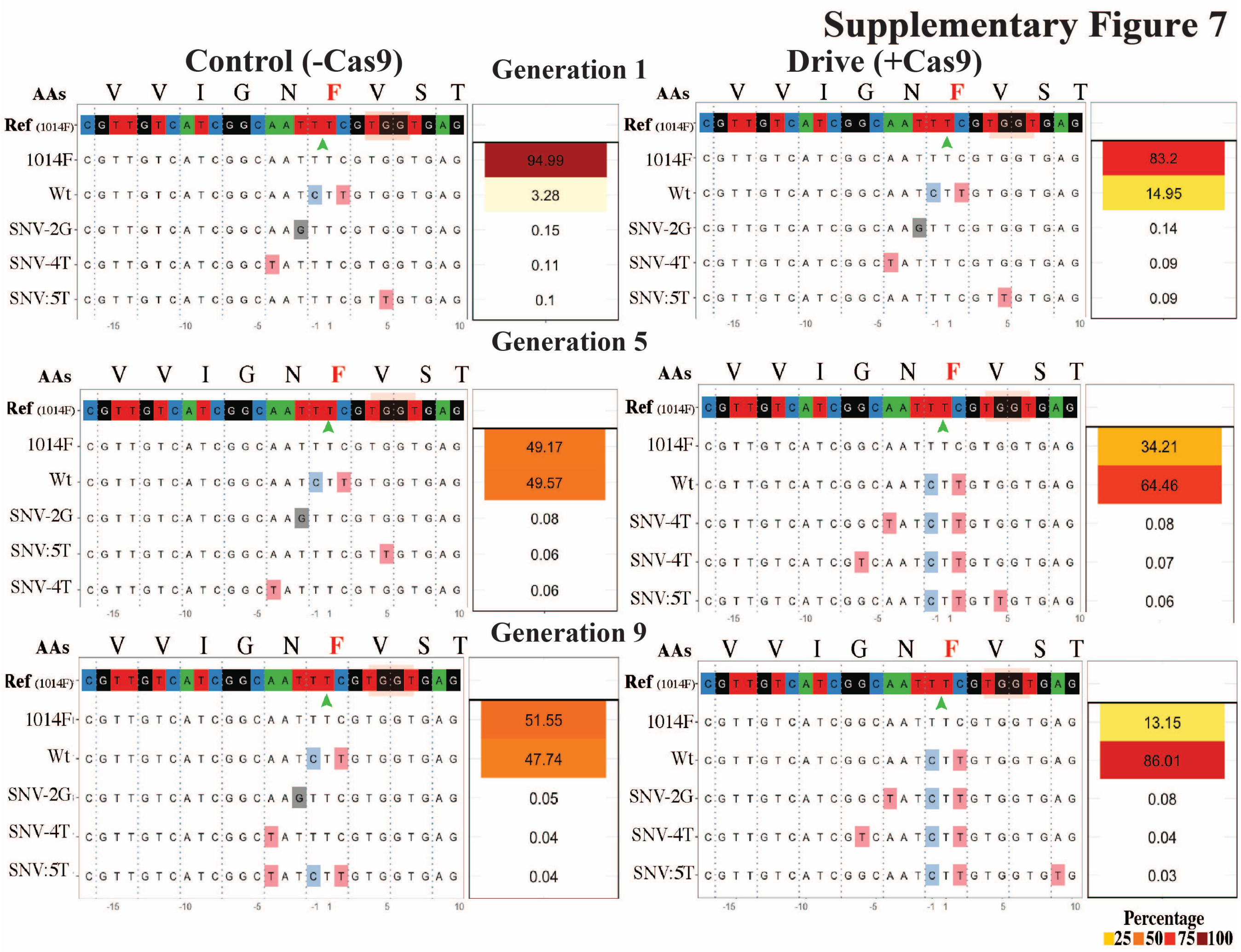

Supplementary Figure 7: Representative Amplicon-sequencing data for control and Drive (+Cas9) cages at generation 1, 5 and 9. A clear decrease in 1014F allele proportions is observed specifically in Drive (+Cas9) cages. Data collected from experiments done as described in Figure 3A.

Supplementary Figure 8

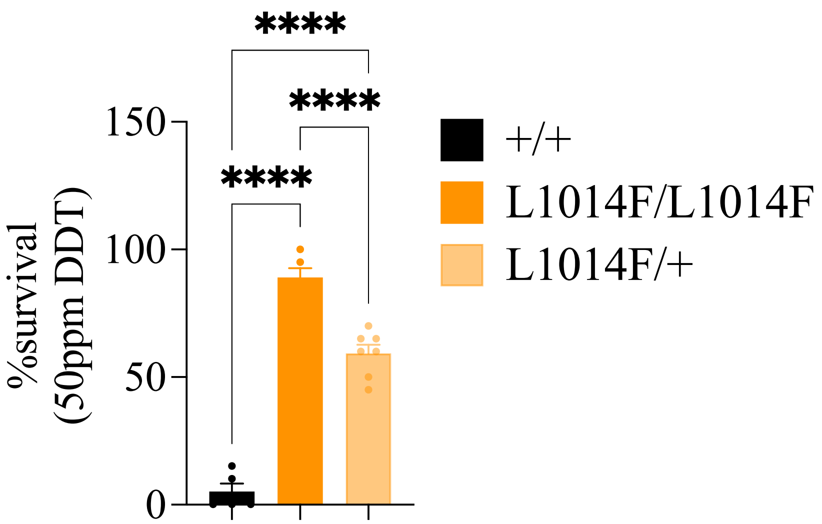

Supplementary Figure 8: Heterozygous L1014F/+ (red) show significant resistance to DDT as compared to wild-type (+/+, grey). The homozygous L1014F/L1014F mutants (orange) show higher resistance as compared to control as well as the heterozygous flies (light orange). N = 5 or more, n = 125 to 150 flies. Mean ± s.e.m is plotted. Data was analyzed 1-Way ANOVA with Sidak multiple comparison tests. **** = p<0.0001

Supplementary Figure 9

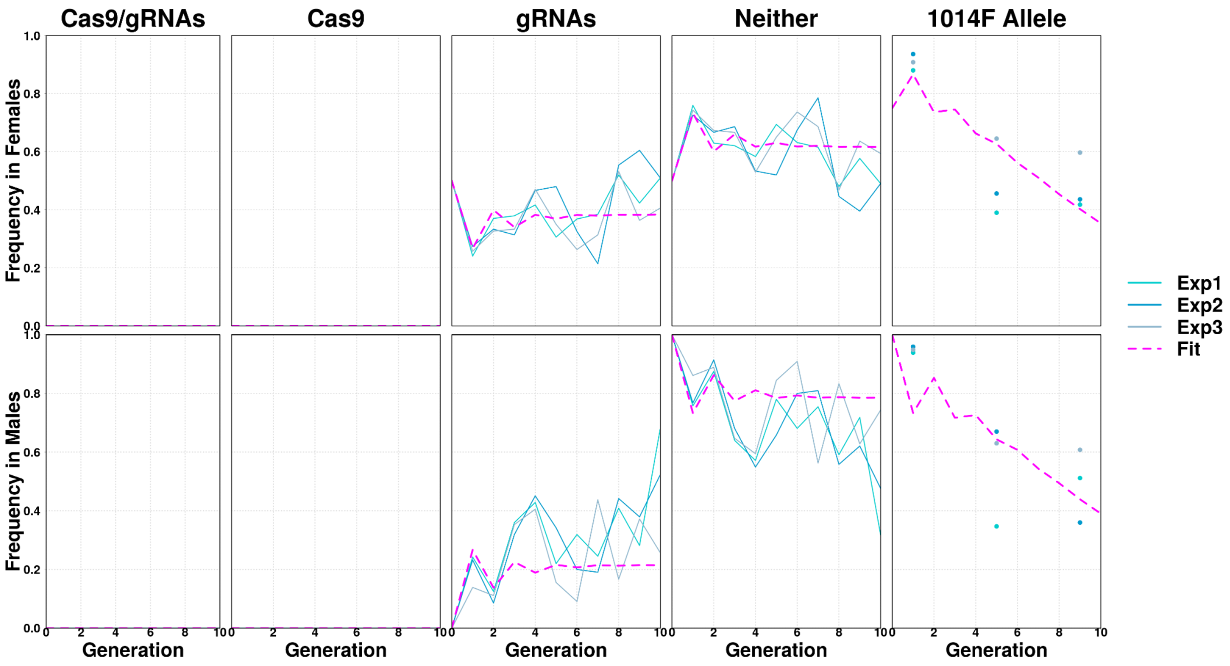

Supplementary Figure 9. Control cage trials and model fit. Observed and model-predicted population dynamics for the gRNAs and 1014F allele. The genotype-to-phenotype mappings are provided in **Supplementary Tables 2-4** and the parameter estimates in **Supplementary Table 5**. Model predictions use a deterministic version of the model, the same version used in the MCMC fitting procedure.

Supplementary Figure 10

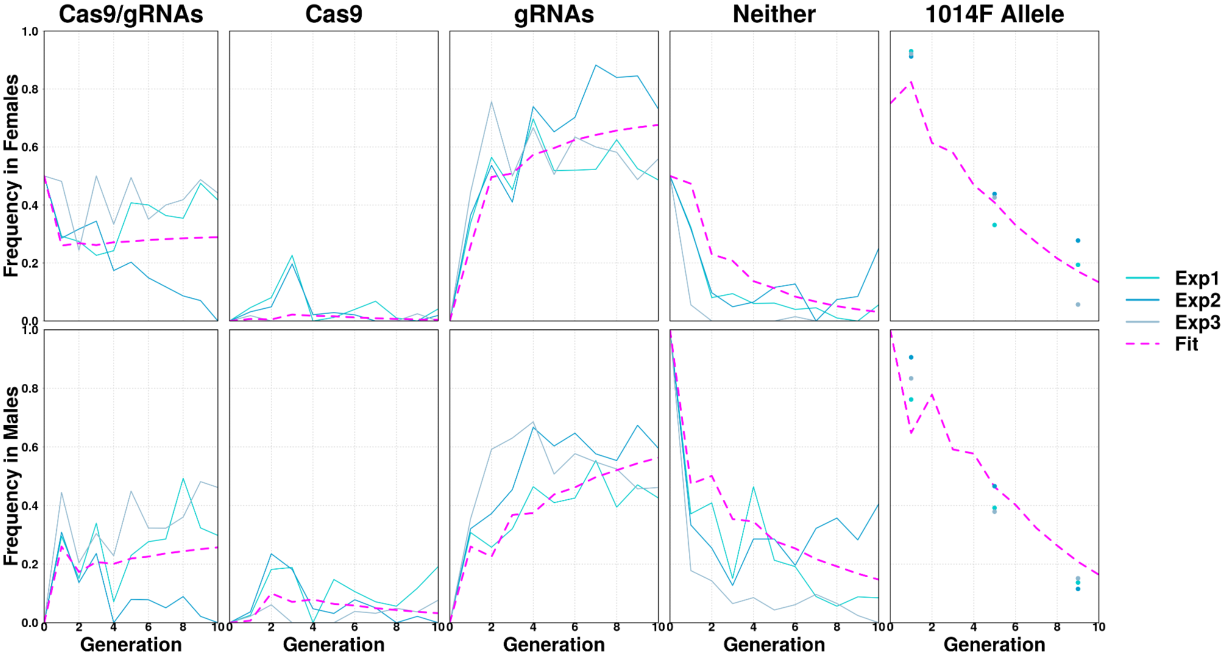

Supplementary Figure 10. Drive cage trials and model fit. Observed and model-predicted population dynamics for the gRNAs and 1014F allele. The genotype-to-phenotype mappings are provided in **Supplementary Tables 2-4** and the parameter estimates in **Supplementary Table 6**. Model predictions use a deterministic version of the model, the same version used in the MCMC fitting procedure.

Supplementary tables

Locus 1 (Chromosome 3)		
**Allele**	**Description**	**Fluorescent Label**
W	Wild-type	Null
C	Cas9	GFP
**Locus 2 (X Chromosome)**		
X	Wild-type	Null
G	gRNAs	DsRed
R	Functional NHEJ	Null
B	Non-functional NHEJ	Null
Y	"Male allele"	Null
**Locus 3 (X Chromosome)**		
**Allele**	**Description**	**Genotype/DDT Label**
X	Wild-type, Major Allele	1014F, DDT-resistant
Z	Wild-type, Minor Allele	1014L, DDT-naive
R	Functional NHEJ	Null
B	Non-functional NHEJ	Null
Y	"Male allele"	Null

Supplementary table 1. Split allelic-drive allele descriptions and fluorescent labels. Locus 1, located on chromosome 3, carries the Cas9 (denoted C, labeled with GFP) allele. When there is no Cas9 present, that locus takes the wild-type (W) designation which has no fluorescent label. As the Cas9 locus is static, it is always inherited in a Mendelian fashion, so resistance alleles are not relevant. Locus 2, located at *yellow* on the X chromosome, contains the gRNA (G, labeled with DsRed) locus. When not present, the wild-type (X) allele and NHEJ alleles all appear without fluorescent labels. NHEJ alleles fall into one of two categories: functional (denoted R for "resistant") alleles, and non-functional (denoted B for "broken") alleles. Locus 3, located at *para* on the X chromosome, contains the major allele (X, the 1014F allele) and

a minor allele (Z, the 1014L allele) in addition to two possible NHEJ alleles, R and B. Locus 3 is not characterized by fluorescent labels, but can be tested via DDT challenge assays or by deep sequencing.

Observed phenotype	Possible Genotypes
None	WWXXXX, WWRXXX, WWBXXX, WWXXXZ, WWRZXX, WWBZXX, WWXRXX, WWRRXX, WWBRXX, WWXBXX, WWRBXX, WWBBXX, WWRXRX, WWBXRX, WWRXXZ, WWRXRZ, WWBZRX, WWRXXR, WWRRRX, WWBRRX, WWRXXB, WWRBRX, WWBBRX, WWBXBX, WWBXXZ, WWBXRZ, WWBXBZ, WWBXXR, WWBXRR, WWBRBX, WWBXXB, WWBXRB, WWBBBX, WWXZXZ, WWRZXZ, WWBZXZ, WWXRXZ, WWRRXZ, WWBRXZ, WWXBXZ, WWRBXZ, WWBBXZ, WWRZRZ, WWBZRZ, WWRZXR, WWRRRZ, WWBRRZ, WWRZXB, WWRBRZ, WWBBRZ, WWBZBZ, WWBZXR, WWBZRR, WWBRBZ, WWBZXB, WWBZRB, WWBBBZ, WWXRXR, WWRRXR, WWBRXR, WWXBXR, WWRBXR, WWBBXR, WWRRRR, WWBRRR, WWRRXB, WWRBRR, WWBBRR, WWBRBR, WWBRXB, WWBRRB, WWBBBR, WWXBXB, WWRBXB, WWBBXB, WWRBRB, WWBBRB, WWBBBB
Green (GFP)	CWXXXX, CWRXXX, CWBXXX, CWXXXZ, CWRZXX, CWBZXX, CWXRXX, CWRRXX, CWBRXX, CWXBXX, CWRBXX, CWBBXX, CWRXRX, CWBXRX, CWRXXZ, CWRXRZ, CWBZRX, CWRXXR, CWRRRX, CWBRRX, CWRXXB, CWRBRX, CWBBRX, CWBXBX, CWBXXZ, CWBXRZ, CWBXBZ, CWBXXR, CWBXRR, CWBRBX, CWBXXB, CWBXRB, CWBBBX, CWXZXZ, CWRZXZ, CWBZXZ, CWXRXZ, CWRRXZ, CWBRXZ, CWXBXZ, CWRBXZ, CWBBXZ, CWRZRZ, CWBZRZ, CWRZXR, CWRRRZ, CWBRRZ, CWRZXB, CWRBRZ, CWBBRZ, CWBZBZ, CWBZXR, CWBZRR, CWBRBZ, CWBZXB, CWBZRB, CWBBBZ, CWXRXR, CWRRXR, CWBRXR, CWXBXR, CWRBXR, CWBBXR, CWRRRR, CWBRRR, CWRRXB, CWRBRR, CWBBRR, CWBRBR, CWBRXB, CWBRRB, CWBBBR, CWXBXB, CWRBXB, CWBBXB, CWRBRB, CWBBRB, CWBBBB, CCXXXX, CCRXXX, CCBXXX, CCXXXZ, CCRZXX, CCBZXX, CCXRXX, CCRRXX, CCBRXX, CCXBXX, CCRBXX, CCBBXX, CCRXRX, CCBXRX, CCRXXZ, CCRXRZ, CCBZRX, CCRXXR, CCRRRX, CCBRRX, CCRXXB, CCRBRX, CCBBRX, CCBXBX, CCBXXZ, CCBXRZ, CCBXBZ, CCBXXR, CCBXRR, CCBRBX, CCBXXB, CCBXRB, CCBBBX, CCXZXZ, CCRZXZ, CCBZXZ, CCXRXZ, CCRRXZ, CCBRXZ, CCXBXZ, CCRBXZ, CCBBXZ, CCRZRZ, CCBZRZ, CCRZXR, CCRRRZ, CCBRRZ, CCRZXB, CCRBRZ, CCBBRZ, CCBZBZ, CCBZXR, CCBZRR, CCBRBZ, CCBZXB, CCBZRB, CCBBBZ, CCXRXR, CCRRXR, CCBRXR, CCXBXR, CCRBXR, CCBBXR, CCRRRR, CCBRRR, CCRRXB, CCRBRR, CCBBRR, CCBRBR, CCBRXB, CCBRRB, CCBBBR, CCXBXB, CCRBXB, CCBBXB, CCRBRB, CCBBRB, CCBBBB
Red (DsRed)	WWGXXX, WWGZXX, WWGRXX, WWGBXX, WWGXGX, WWGXRX, WWBXGX, WWGXXZ, WWGXGZ, WWGXRZ, WWBZGX, WWGXXR, WWGRGX, WWGXRR, WWBRGX, WWGXXB, WWGBGX, WWGXRB, WWBBGX, WWGZRX, WWGRRX, WWGBRX, WWBXGZ, WWBXGR, WWBXGB, WWGZXZ, WWGRXZ, WWGBXZ, WWGZGZ, WWGZRZ, WWBZGZ, WWGZXR, WWGRGZ, WWGZRR, WWBRGZ, WWGZXB, WWGBGZ, WWGZRB, WWBBGZ, WWGRRZ, WWGBRZ, WWBZGR, WWBZGB, WWGRXR, WWGBXR, WWGRGR, WWGRRR, WWBRGR, WWGRXB, WWGBGR, WWGRRB, WWBBGR, WWGBRR, WWBRGB, WWGBXB, WWGBGB, WWGBRB, WWBBGB
Both (GFP/DsRed)	CWGXXX, CWGZXX, CWGRXX, CWGBXX, CWGXGX, CWGXRX, CWBXGX, CWGXXZ, CWGXGZ, CWGXRZ, CWBZGX, CWGXXR, CWGRGX, CWGXRR, CWBRGX, CWGXXB, CWGBGX, CWGXRB, CWBBGX, CWGZRX, CWGRRX, CWGBRX, CWBXGZ, CWBXGR, CWBXGB, CWGZXZ, CWGRXZ, CWGBXZ, CWGZGZ, CWGZRZ, CWBZGZ, CWGZXR, CWGRGZ, CWGZRR, CWBRGZ, CWGZXB, CWGBGZ, CWGZRB, CWBBGZ, CWGRRZ, CWGBRZ, CWBZGR, CWBZGB, CWGRXR, CWGBXR, CWGRGR, CWGRRR, CWBRGR, CWGRXB, CWGBGR, CWGRRB, CWBBGR, CWGBRR, CWBRGB, CWGBXB, CWGBGB, CWGBRB, CWBBGB, CCGXXX, CCGZXX, CCGRXX, CCGBXX, CCGXGX, CCGXRX, CCBXGX, CCGXXZ, CCGXGZ, CCGXRZ, CCBZGX, CCGXXR, CCGRGX, CCGXRR, CCBRGX, CCGXXB, CCGBGX, CCGXRB, CCBBGX, CCGZRX, CCGRRX, CCGBRX, CCBXGZ, CCBXGR, CCBXGB, CCGZXZ, CCGRXZ, CCGBXZ, CCGZGZ, CCGZRZ, CCBZGZ, CCGZXR, CCGRGZ, CCGZRR, CCBRGZ, CCGZXB, CCGBGZ, CCGZRB, CCBBGZ, CCGRRZ, CCGBRZ, CCBZGR, CCBZGB, CCGRXR, CCGBXR, CCGRGR, CCGRRR, CCBRGR, CCGRXB, CCGBGR, CCGRRB, CCBBGR, CCGBRR, CCBRGB, CCGBXB, CCGBGB, CCGBRB, CCBBGB

Supplementary table 2: Female split allelic-drive phenotype-to-genotype mappings. As there are three fluorescent labels (GFP, DsRed, and none, see **Supplementary Tables 1**) in loci 1 and 2, there are 4 possible phenotypes. These phenotypes provide an indication of the genotype of an individual, as each

genotype generates a specific phenotype. From that, we can classify all genotypes as 1 of 4 phenotypes observed in the experiments. Within genotypes, the loci are organized as 112323, where each number corresponds to the locus labels. Locus 1 is autosomal and independent of everything. Loci 2 and 3, *yellow* and *para*, are both on the X chromosome and could possibly demonstrate linkage (the expectation is complete independence given their genetic distance). Therefore, loci 2 and 3 are associated with each other in the genotype labeling. (For clarity, complete independence was used for this modeling).

Observed phenotype	Possible Genotypes
None	WWXXYY, WWRXYY, WWBXYY, WWXZYY, WWRZYY, WWBZYY, WWXRYY, WWRRYY, WWBRYY, WWXBYY, WWRBYY, WWBBYY
Green (GFP)	CWXXYY, CWRXYY, CWBXYY, CWXZYY, CWRZYY, CWBZYY, CWXRYY, CWRRYY, CWBRYY, CWXBYY, CWRBYY, CWBBYY, CCXXYY, CCRXYY, CCBXYY, CCXZYY, CCRZYY, CCBZYY, CCXRYY, CCRRYY, CCBRYY, CCXBYY, CCRBYY, CCBBYY
Red (DsRed)	WWGXYY, WWGZYY, WWGRYY, WWGBYY
Both (GFP/DsRed)	CWGXYY, CWGZYY, CWGRYY, CWGBYY, CCGXYY, CCGZYY, CCGRYY, CCGBYY

Supplementary table 3. Male split allelic-drive phenotype-to-genotype mappings. As there are three fluorescent labels (GFP, DsRed, and none, see **Supplementary Table 1**) in loci 1 and 2, there are 4 possible phenotypes. These phenotypes provide an indication of the genotype of an individual, as each genotype generates a specific phenotype. From that, we can classify all genotypes as 1 of 4 phenotypes observed in the experiments. Within genotypes, the loci are organized as 112323, where each number corresponds to the locus labels. Locus 1 is autosomal and independent of everything. Loci 2 and 3, *yellow* and *para*, are both on the X chromosome and could possibly demonstrate linkage (the expectation is complete independence given their genetic distance). Therefore, loci 2 and 3 are associated with each other in the genotype labeling. To denote males, we made one set of loci 2 and 3 be Y, hence all genotypes being 1123YY. (For clarity, complete independence on the X chromosome was used for this modeling, male Y alleles were kept together at all times).

Sex	Genotype Counts
Female	WWXXXX, WWGXXX, WWRXXX, WWBXXX, WWXXXZ, WWGZXX, WWRZXX, WWBZXX, WWXRXX, WWGRXX, WWRRXX, WWBRXX, WWXBXX, WWGBXX, WWRBXX, WWBBXX, WWGXGX, WWGXRX, WWBXGX, WWGXXZ, WWGXGZ, WWGXRZ, WWBZGX, WWGXXR, WWGRGX, WWGXRR, WWBRGX, WWGXXB, WWGBGX, WWGXRB, WWBBGX, WWRXRX, WWBXRX, WWRXXZ, WWGZRX, WWRXRZ, WWBZRX, WWRXXR, WWGRRX, WWRRRX, WWBRRX, WWRXXB, WWGBRX, WWRBRX, WWBBRX, WWBXBX, WWBXXZ, WWBXGZ, WWBXRZ, WWBXBZ, WWBXXR, WWBXGR, WWBXRR, WWBRBX, WWBXXB, WWBXGB, WWBXRB, WWBBBX, CWXXXX, CWGXXX, CWRXXX, CWBXXX, CWXXXZ, CWGZXX, CWRZXX, CWBZXX, CWXRXX, CWGRXX, CWRRXX, CWBRXX, CWXBXX, CWGBXX, CWRBXX, CWBBXX, CWGXGX, CWGXRX, CWBXGX, CWGXXZ, CWGXGZ, CWGXRZ, CWBZGX, CWGXXR, CWGRGX, CWGXRR, CWBRGX, CWGXXB, CWGBGX, CWGXRB, CWBBGX, CWRXRX, CWBXRX, CWRXXZ, CWGZRX, CWRXRZ, CWBZRX, CWRXXR, CWGRRX, CWRRRX, CWBRRX, CWRXXB, CWGBRX, CWRBRX, CWBBRX, CWBXBX, CWBXXZ, CWBXGZ, CWBXRZ, CWBXBZ, CWBXXR, CWBXGR, CWBXRR, CWBRBX, CWBXXB, CWBXGB, CWBXRB, CWBBBX, CCXXXX, CCGXXX, CCRXXX, CCBXXX, CCXXXZ, CCGZXX, CCRZXX, CCBZXX, CCXRXX, CCGRXX, CCRRXX, CCBRXX, CCXBXX, CCGBXX, CCRBXX, CCBBXX, CCGXGX, CCGXRX, CCBXGX, CCGXXZ, CCGXGZ, CCGXRZ, CCBZGX, CCGXXR, CCGRGX, CCGXRR, CCBRGX, CCGXXB, CCGBGX, CCGXRB, CCBBGX, CCRXRX, CCBXRX, CCRXXZ, CCGZRX, CCRXRZ, CCBZRX, CCRXXR, CCGRRX, CCRRRX, CCBRRX, CCRXXB, CCGBRX, CCRBRX, CCBBRX, CCBXBX, CCBXXZ, CCBXGZ, CCBXRZ, CCBXBZ, CCBXXR, CCBXGR, CCBXRR, CCBRBX, CCBXXB, CCBXGB, CCBXRB, CCBBBX, WWXXXX, WWGXXX, WWRXXX, WWBXXX, WWGXGX, WWGXRX, WWBXGX, WWRXRX, WWBXRX, WWBXBX, CWXXXX, CWGXXX, CWRXXX, CWBXXX, CWGXGX, CWGXRX, CWBXGX, CWRXRX, CWBXRX, CWBXBX, CCXXXX, CCGXXX, CCRXXX, CCBXXX, CCGXGX, CCGXRX, CCBXGX, CCRXRX, CCBXRX, CCBXBX
Male	WWXXYY, WWGXYY, WWRXYY, WWBXYY, CWXXYY, CWGXYY, CWRXYY, CWBXYY, CCXXYY, CCGXYY, CCRXYY, CCBXYY

Supplementary table 4. 1014F genotype counts for deep sequencing mapping. There is no fluorescent label associated with the *para* locus, as the intention is to study allelic conversion from the 1014F allele to 1014L. Therefore, deep sequencing of generations 1, 5, and 9 was performed to obtain the 1014F allele frequency at those three time points. The genotype counts in the table define how 1014F allele frequencies were calculated from the simulations for purposes of model fitting and plotting. As 1014L and NHEJ alleles were not quantified independently, simply as "not 1014F", simulations simply used 1 minus the above frequencies to generate them.

Parameters		Differential Evolution MCMC Parameter Estimates			
**Label**	**Description**	**MAP**	**2.5% quantile**	**Median**	**97.5% quantile**
c_F^Y	Female Cleavage Rate, *yellow*	0			
$p_F^{Y,HDR}$	Female HDR Rate, *yellow*	0			
$p_F^{Y,NHEJ}$	Female Functional NHEJ Rate, *yellow*	0			
c_F^P	Female Cleavage Rate, *para*	0			
$p_F^{P,HDR}$	Female HDR Rate, *para*	0			
c_M^P	Male Cleavage Rate, *para*	0			
d^Y	Female Deposition Rate, *yellow*	0			
d^P	Female Deposition Rate, *para*	0			
s_F^F	Female Fitness Cost, One 1014F	0.00	0.00	0.01	0.03
s_F^{FF}	Female Fitness Cost, Two 1014F	0.13	0.04	0.13	0.20
s_M^F	Male Fitness Cost, One 1014F	0.28	0.11	0.27	0.43

Supplementary table 5. Parameter estimates, control cages. Parameters were estimated by running the log-likelihood (above) through a differential evolution MCMC algorithm. The maximum a posteriori estimate (MAP) is the point that appears most often, and is the best point estimate for our parameters. Additionally, the mean and 95% credible intervals are provided. Only fitness cost parameters were estimated from the control cages, therefore all drive-related parameters are, by definition, 0.

Parameters		Differential Evolution MCMC Parameter Estimates			
**Label**	**Description**	**MAP**	**2.5% quantile**	**Median**	**97.5% quantile**
c_F^Y	Female Cleavage Rate, *yellow*	0.95	0.94	0.97	1.00
$p_F^{Y,HDR}$	Female HDR Rate, *yellow*	1.00	0.96	0.99	1.00
$p_F^{Y,NHEJ}$	Female Functional NHEJ Rate, *yellow*	0.33	0.03	0.50	0.97
c_F^P	Female Cleavage Rate, *para*	0.32	0.30	0.33	0.36
$p_F^{P,HDR}$	Female HDR Rate, *para*	1.00	0.97	0.99	1.00
c_M^P	Male Cleavage Rate, *para*	1.00	0.88	0.97	1.00
d^Y	Female Deposition Rate, *yellow*	0.01	0.00	0.01	0.05
d^P	Female Deposition Rate, *para*	0.00	0.00	0.00	0.01
s_F^F	Female Fitness Cost, One 1014F	0.00			
s_F^{FF}	Female Fitness Cost, Two 1014F	0.13			
s_M^F	Male Fitness Cost, One 1014F	0.28			

Supplementary table 6: Parameter estimates, drive cages. Parameters were estimated by running the log-likelihood (above) through a differential evolution MCMC algorithm. The maximum a posteriori estimate (MAP) is the point that appears most often, and is the best point estimate for our parameters. Additionally, the mean and 95% credible intervals are provided. For consistency, fitness costs were fixed at the MAP values estimated from the control cages.

Supplementary note 1

Model fitting was carried out using a likelihood-based MCMC algorithm to sample from parameter space and explore parameter distributions. Under laboratory cage conditions, we assume discrete generations and a randomly mixing population. We model the population dynamics of allelic-drive as a multiplexed split-drive system. First, we describe the phenotype-genotype mapping for the system, then the likelihood calculation involving phenotype and deep sequencing data, and finally the generation of stochastic simulations.

This allelic-drive is implemented as a split-drive system, with the Cas9 construct on chromosome 3 and labeled with GFP. We implement this using 2 alleles, W for wild-type and C for Cas9 alleles. The gRNAs are located in the *yellow* gene on the X chromosome and labeled with DsRed. As there is the possibility of CRISPR-mediated cleavage/repair at this locus, we implement 4 alleles: X for wild-type, G for gRNAs, R for in-frame or otherwise functional alleles (elsewhere referred to as "R1" alleles), and B for out-of-frame or otherwise broken alleles (elsewhere referred to as "R2" alleles). R and B alleles do not need to be defined as such, they can be used to represent any 2 NHEJ alleles with differing fitness effects. The wild-type (X) for this locus carries a mutation that disrupts *yellow* function, so the entire population is *yellow*- to avoid assortative mating impacts, but does not impact cleavage at the target site.

The *para* locus is used for allelic-drive, where the 1014F allele is DDT resistant and the 1014L allele is DDT naive. We define the 1014F allele as wild-type, X, as it is the major allele in the population. We define the 1014L allele as a minor allele, Z. Because there is also CRISPR-mediated cleavage/repair at this locus, we use R and B alleles here as well, following the same definitions as above. Additionally, as males are monoploid for the X chromosome, we denote a Y allele for the *yellow* and *para* locus, which does not participate in cleavage/homing events, as a placeholder for ease of use by the modeling team. These alleles, and their corresponding marker phenotypes, are summarized in Supplementary Table1. As flies are diploid, this implies 3 unique and viable genotypes at locus 1, the autosomal locus. For the *yellow* and *para* loci, they are unlinked but both on the X chromosome, so there are 16 distinct X chromosomes (and 1 "Y" chromosome). Combining the autosomes independently with the sex chromosomes, we have 408 distinct female genotypes and 48 distinct male genotypes. Based on fluorescent labels and deep sequencing, phenotypes/allele frequencies are defined for all genotypes, as summarized in Supplementary Table 2-4.

Given the large number of possible mating pairs, it is not feasible to show the complete equations for the next generation genotype frequencies. Instead, we define a set of rules describing offspring genotype

frequencies for all mating classes, and explain their application using examples for each mating class. While all crosses accept sex-specific parameters, the general equations are agnostic to sex.

The simplest inheritance scenarios are those where there is no gene drive involved. As this is a split-drive system, both the Cas9 and gRNA alleles must be present for homing to occur. Individuals without at least one copy of each of the Cas9 and gRNA alleles therefore abide by Mendelian inheritance rules. The only exception to this comes from female deposition of Cas9 into early zygotes, impacting the contribution of male sperm. If the mother is able to produce loaded Cas9-gRNA RnP complexes, X chromosomes contributed by fathers are subject to cleavage at a rate d^Y at the *yellow* locus and d^P at the *para* locus. As there is no HDR in male flies, and *para* is a conserved gene, all cleavage events are assumed to generate out-of-frame NHEJ alleles. Additionally, the high variance estimated for these events precluded robust quantification of HDR and in-frame NHEJ rates, or determination of copy-number dependence.

In females, when at least one each of the Cas9 and gRNA alleles are present in an individual, the pieces combine to promote Cas9-mediated cleavage and repair. We do not fit copy number-dependent parameters in this analysis, therefore having one Cas9 allele provides the same cleavage rate as two copies. In the presence of both Cas9 and gRNA alleles, a fraction, c_F^Y of wild-type *yellow* alleles and c_F^P of 1014F *para* alleles are cleaved, while the remaining $1 - c_F^{\{Y,P\}}$ remain wild-type. Given cleavage, accurate HDR occurs at a rate $p_F^{Y,HDR}$ at *yellow* or $p_F^{P,HDR}$ at *para*, while the remaining $1 - p_F^{\{Y,P\},HDR}$ alleles undergoing some form of non-homologous end-joining (NHEJ). At *yellow*, a fraction, $p_F^{Y,NHEJ}$, of alleles that are cleaved but do not undergo HDR are repaired in-frame, creating functional alleles, while the remaining $1 - p_F^{Y,NHEJ}$ are repaired out-of-frame, generating nonsense mutations and creating non-functional alleles. As *para* is an essential gene, any alleles that were cleaved but did not undergo HDR are assumed to become out-of-frame NHEJ alleles.

Male CRISPR-mediated cleavage occurs under the same conditions as females, with the wrinkle that they possess only one X chromosome. Therefore, no cleavage or repair is possible at the *yellow* locus. At the *para* locus, cleavage may occur at a rate c_M^P. As there is no sister chromatid for allelic conversion, and male flies do not perform HDR, only NHEJ allele generation occurs. *Para* is an essential gene, so it is assumed that all cleaved alleles become out-of-frame NHEJ alleles. Parameters for male and female allelic-drive are listed in Supplementary Table 5 and Supplementary Table 6, including a short explanation of each parameter and maximum likelihood parameter estimates from model fitting.

In addition to parameters determining inheritance bias, the gene drive system is associated with fitness costs due to its several components. We disregard assortative mating issues because all experiments were performed in a *yellow-* background. Prior experimental work demonstrated negligible fitness effects from the Cas9 or gRNA alleles, therefore we focus on effects from the *para* locus. As *para* is an essential gene, but also haplosufficient, anytime two NHEJ alleles are found together at this locus the individual is unviable at birth. Additionally, the 1014F allele is thought to be deleterious compared to the 1014L allele. Thus, we define copy-number dependent fitness costs in females, s_F^F and s_F^{FF}, for possession of one or two 1014F alleles, and require that the homozygote have a greater fitness cost than the heterozygote. As males are monoploid in X, we define a single cost, s_M^F, for possession of the 1014F allele, and require it to be equal or greater than the cost on females (since there is no rescue allele). Fitness is defined relative to wild-type organisms. As we apply multiple fitness costs additively, we must ensure that resulting cumulative fitness costs are between 0 and 1. We achieved this by truncating the total cost for an individual at 100%, using $min(cost, 1)$. This is intuitive biologically, as having two costly alleles implies that the organism experiences the fitness impact of both; however, it ignores any synergistic or nonlinear effects resulting from combining several costs in one organism.

Considering these inheritance biases and fitness costs, we calculate the expected genotype frequencies, denoted p_k^x for genotype x in generation k, or allele frequencies, denoted f_k^x for genotype x in generation k, in each generation. Genotype and allele frequencies are normalized at each generation to ensure they sum to 1.

The likelihood of the population cage data was then calculated by assuming a multinomial distribution of individuals having each sex and marker phenotype, and by using the model predictions to generate expected proportions for each set of parameter values. I.e., by calculating the log likelihood,

$$logL(\theta) \propto \sum_{i=1}^{3} \sum_{k=1}^{n_i} \left(\sum_{P \in \{P_F, P_M\}} N_{i,k}^P \cdot log(p_k^P(\theta)) + a \cdot \sum_{G \in \{G_F, G_M\}} F_{i,k}^G \cdot log(f_k^G(\theta)) \right).$$

The log-likelihood is a summation over the three replicates, *i*, over each generation, *k*, and over a composite function containing genotype and allele frequencies. $N_{i,k}^P$ denotes the number of individuals at generation k in experiment i, containing female, P_F, or male, P_M, phenotypes collectively. $F_{i,k}^G$ denotes the allele frequencies at the *para* locus at generation k in experiment i, containing female, G_F, or male, G_M, allele frequencies. Note, these frequencies are only defined for generations 1, 5, and 9, and are 0 otherwise. a is a constant to ensure phenotype and allele calculations are within the same order of magnitude for improved

MCMC estimation. Possible phenotypes with corresponding genotype-phenotype mappings are given in Supplementary Table 2, Supplementary Table 3, while genotype frequency mappings for the *para* locus are found in Supplementary Table 4. All cage trials were run for 10 generations, and expected phenotype frequencies, $p_k^P(\theta)$, and allele frequencies, $f_k^G(\theta)$, at generation k are dependent on the model parameters, $\theta = \{c_F^Y, p_F^{Y,HDR}, p_F^{Y,NHEJ}, c_F^P, p_F^{P,HDR}, c_M^P, d^Y, d^P, s_F^F, s_F^{FF}, s_M^F\}$. See Supplementary Table 5 and Supplementary Table 6 for parameter definitions and estimates for each trial.

Experiments were seeded with a mixture of wild-type and heterozygous gene drive flies. This mixture was dependent on the cage design:

A) The control cages began with 100 flies, equally split between sexes. All males began wild-type. Half the females began wild-type while the other half began cis-heterozygous for gRNA and 1014L. This corresponds to genotypes "WWXXYY" for males (see Supplementary Table 3) or "WWXXXX" and "WWGZXX" for females (see Supplementary Table 2).
B) The drive cages began with 100 flies, equally split between sexes. All males began wild-type. Half the females began wild-type while the other half began cis-heterozygous for gRNA and 1014L and trans-heterozygous for Cas9. This corresponds to genotypes "WWXXYY" for males (see Supplementary Table 3) or "WWXXXX" and "CWGZXX" for females (see Supplementary Table 2).

Models were fitted using a Differential-Evolution MCMC procedure with snooker updating from the R package BayesianTools (Hartig *et al.*, 2019). The fitting procedure implemented uniform priors and the final fits were a single run of 1.5 million iterations - 500 thousand iterations per chain. The estimated parameters, along with some statistics about each parameter, are included in Supplementary Table 5 and Supplementary Table 6. Each fit is visualized with the corresponding cage trial data in Supplementary Figure 1 and Supplementary Figure 2

Supplementary Note 2

YCCP Plasmid sequence

TTGCGTATTGGGCGCTCTTCCGCTTCCTCGCTCACTGACTCGCTGCGCTCGGTCGTTCGGCTG
CGGCGAGCGGTATCAGCTCACTCAAAGGCGGTAATACGGTTATCCACAGAATCAGGGGATA
ACGCAGGAAAGAACATGTGAGCAAAAGGCCAGCAAAAGGCCAGGAACCGTAAAAAGGCCG
CGTTGCTGGCGTTTTTCCATAGGCTCCGCCCCCCTGACGAGCATCACAAAAATCGACGCTCA
AGTCAGAGGTGGCGAAACCCGACAGGACTATAAAGATACCAGGCGTTTCCCCCTGGAAGCT
CCCTCGTGCGCTCTCCTGTTCCGACCCTGCCGCTTACCGGATACCTGTCCGCCTTTCTCCCTTC
GGGAAGCGTGGCGCTTTCTCATAGCTCACGCTGTAGGTATCTCAGTTCGGTGTAGGTCGTTC
GCTCCAAGCTGGGCTGTGTGCACGAACCCCCCGTTCAGCCCGACCGCTGCGCCTTATCCGGT
AACTATCGTCTTGAGTCCAACCCGGTAAGACACGACTTATCGCCACTGGCAGCAGCCACTGG
TAACAGGATTAGCAGAGCGAGGTATGTAGGCGGTGCTACAGAGTTCTTGAAGTGGTGGCCT
AACTACGGCTACACTAGAAGAACAGTATTTGGTATCTGCGCTCTGCTGAAGCCAGTTACCTT
CGGAAAAAGAGTTGGTAGCTCTTGATCCGGCAAACAAACCACCGCTGGTAGCGGTGGTTTTT
TTGTTTGCAAGCAGCAGATTACGCGCAGAAAAAAAGGATCTCAAGAAGATCCTTTGATCTTT
TCTACGGGGTCTGACGCTCAGTGGAACGAAAACTCACGTTAAGGGATTTTGGTCATGAGATT
ATCAAAAAGGATCTTCACCTAGATCCTTTTAAATTAAAAATGAAGTTTTAAATCAATCTAAA
GTATATATGAGTAAACTTGGTCTGACAGTTACCAATGCTTAATCAGTGAGGCACCTATCTCA
GCGATCTGTCTATTTCGTTCATCCATAGTTGCCTGACTCCCCGTCGTGTAGATAACTACGATA
CGGGAGGGCTTACCATCTGGCCCCAGTGCTGCAATGATACCGCGAGACCCACGCTCACCGGC
TCCAGATTTATCAGCAATAAACCAGCCAGCCGGAAGGGCCGAGCGCAGAAGTGGTCCTGCA
ACTTTATCCGCCTCCATCCAGTCTATTAATTGTTGCCGGGAAGCTAGAGTAAGTAGTTCGCCA
GTTAATAGTTTGCGCAACGTTGTTGCCATTGCTACAGGCATCGTGGTGTCACGCTCGTCGTTT
GGTATGGCTTCATTCAGCTCCGGTTCCCAACGATCAAGGCGAGTTACATGATCCCCCATGTT
GTGCAAAAAAGCGGTTAGCTCCTTCGGTCCTCCGATCGTTGTCAGAAGTAAGTTGGCCGCAG
TGTTATCACTCATGGTTATGGCAGCACTGCATAATTCTCTTACTGTCATGCCATCCGTAAGAT
GCTTTTCTGTGACTGGTGAGTACTCAACCAAGTCATTCTGAGAATAGTGTATGCGGCGACCG
AGTTGCTCTTGCCCGGCGTCAATACGGGATAATACCGCGCCACATAGCAGAACTTTAAAAGT
GCTCATCATTGGAAAACGTTCTTCGGGGCGAAAACTCTCAAGGATCTTACCGCTGTTGAGAT
CCAGTTCGATGTAACCCACTCGTGCACCCAACTGATCTTCAGCATCTTTTACTTTCACCAGCG
TTTCTGGGTGAGCAAAAACAGGAAGGCAAAATGCCGCAAAAAAGGGAATAAGGGCGACAC
GGAAATGTTGAATACTCATACTCTTCCTTTTTCAATTCAGAAGAACTCGTCAAGAAGGCGAT
AGAAGGCGATGCGCTGCGAATCGGGAGCGGCGATACCGTAAAGCACGAGGAAGCGGTCAG
CCCATTCGCCGCCAAGCTCTTCAGCAATATCACGGGTAGCCAACGCTATGTCCTGATAGCGG

TCCGCCACACCCAGCCGGCCACAGTCGATGAATCCAGAAAAGCGGCCATTTTCCACCATGAT
ATTCGGCAAGCAGGCATCGCCATGGGTCACGACGAGATCCTCGCCGTCGGGCATGCGCGCCT
TGAGCCTGGCGAACAGTTCGGCTGGCGCGAGCCCCTGATGCTCTTCGTCCAGATCATCCTGA
TCGACAAGACCGGCTTCCATCCGAGTACGTGCTCGCTCGATGCGATGTTTCGCTTGGTGGTC
GAATGGGCAGGTAGCCGGATCAAGCGTATGCAGCCGCCGCATTGCATCAGCCATGATGGAT
ACTTTCTCGGCAGGAGCAAGGTGGGATGACAGGAGATCCTGCCCCGGCACTTCGCCCAATA
GCAGCCAGTCCCTTCCCGCTTCAGTGACAACGTCGAGCACAGCTGCGCAAGGAACGCCCGTC
GTGGCCAGCCACGATAGCCGCGCTGCCTCGTCCTGCAGTTCATTCAGGGCACCGGACAGGTC
GGTCTTGACAAAAAGAACCGGGCGCCCCTGCGCTGACAGCCGGAACACGGCGGCATCAGAG
CAGCCGATTGTCTGTTGTGCCCAGTCATAGCCGAATAGCCTCTCCACCCAAGCGGCCGGAGA
ACCTGCGTGCAATCCATCTTGTTCAATCATGCGAAACGATCCTCATCCTGTCTCTTGATCAGA
TCTTGATCCCCTGCGCCATCAGATCCTTGGCGGCAAGAAAGCCATCCAGTTTACTTTGCAGG
GCTTCCCAACCTTACCAGAGGGCGCCCCAGCTGGCAATTCCGGTTCGCTTGCTGTCCATAAA
ACCGCCCAGTCTAGCTATCGCCATGTAAGCCCACTGCAAGCTACCTGCTTTCTCTTTGCGCTT
GCGTTTTCCCTTGTCCAGATAGCCCAGTAGCTGACATTCATCCGGGGTCAGCACCGTTTCTGC
GGACTGGCTTTCTACGTGTTCCGCTTCCTTTAGCAGCCCTTGCGCCCTGAATTTTGTTAAAAT
TCGCGTTAAATTTTTGTTAAATCAGCTCATTTTTTAACCAATAGGCCGAAATCGGCAAAATCC
CTTATAAATCAAAAGAATAGACCGAGATAGGGTTGAGTGTTGTTCCAGTTTGGAACAAGAGT
CCACTATTAAAGAACGTGGACTCCAACGTCAAAGGGCGAAAAACCGTCTATCAGGGCGATG
GCCCACTACGTGAACCATCACCCTAATCAAGTTTTTTGGGGTCGAGGTGCCGTAAAGCACTA
AATCGGAACCCTAAAGGGAGCCCCCGATTTAGAGCTTGACGGGGAAAGCCGGCGAACGTGG
CGAGAAAGGAAGGGAAGAAAGCGAAAGGAGCGGGCGCTAGGGCGCTGGCAAGTGTAGCGG
TCACGCTGCGCGTAACCACCACACCCGCCGCGCTTAATGCGCCGCTACAGGGCGCGTCCATT
CGCCATTCAGGCTGCGCAACTGTTGGGAAGGGCGATCGGTGCGGGCCTCTTCGCTATTACGC
CAGCTGGCGAAAGGGGGATGTGCTGCAAGGCGATTAAGTTGGGTAACGCCAGGGTTTTCCC
AGTCACGACGTTGTAAAACGACGGCCAGTGAATTGTAATACGACTCACTATAGGGCGAATT
GGGCCCTCTAGATGCATGCTCGAGCGGCCGCCAGTGTGATGGATATCTGCAGAATTCGCCCT
Tgttgcgaggttttaggactgaaagagcacatgtcaaaatataaatttgttcaaatactttatatttgactgaattagattgttattttaaaagttatgaattaaata
aagattgaaaggtgcattatgctcaaatgtatatttatcgcaacccccggttactttgtaaagcaaaaacgcctggtttgatttttaagaagatgggtcggtaa
atcgataaaagctatattttctggtcgttgcagtctcactcgcctgctataaaaacattaaaagttcccagaaacaataaatgtctttaaattcaattaacgaaga
aataaagaaggaaaagaactggagcggaaatcggtcgaaatactgccaatggccacatatacatttaacagcgatatatggtatacatattgataatgatgt
cagacgcaattgcttcagacggctaatgacatcgcaaattgcacgcaacttgcaatagtgccaattatgactgaagtacatatagccggggatcttttaaca
taaacttccagtagatgtacaagcagaaaaaagagccattagcacggcagttaccattgcttatgattccttgtgtccaaaataatgacaaataggtatataa
ataattaaatgccaaacataagcgattctaatttacctttacatctgtatgcatttacatattatccagaaaacagacagcgataacttgcaacattgcttagtata

ataatccaaagaaggaatttaggcagaaattccagttaattaaatattcaaaacaaactttatttagtgcctcaataatagtttggccctgctaattctcctatttta
ttttttagggattccggccactctgacctatataaacatggaccgcagtttgacgggttcaccggagctaattccgtatccagattggcgctcaaatacagct
ggagattgcgccaacagtattaccactgcctaccgcattaaagtggatgagtgtggtcggctgtgggttttggacactggaagacgtcATTTTCA
ACGTCCTCGATAGTATAGTGGTTAGTATCCCCGCCTGTCACGCGGGAGACCGGGGTTCAATT
CCCCGTCGGGGAGAATCTGTGATTCTTTTTTTTTTTCTTTTACTTTGTTATATAAACAATTTTT
GTTTTAATTGAATCTAATTTGCCATTGCTTTTAGGAATCTCAGGCATCCAGCAAGCGTTTGTC
CGCCGAATCGCCCATCAGTGAAGAAGATCCTGTGGCGGCTACGAAAATCTCCCCGGCCATGT
CGGCCTCCACCTCCAGCGAAAAACCCATCAGCGAGCTGGCCACCTCTGTGCTGACCCACCGC
TTTCCAGACTCCACCTCCTCACCCGGCGAACATGGCCTTGGACGAATGCAGTTGTCGATCCG
CTACAGCGCCCAGCGTCAAAAACTAGACGTGACCATACACAAAATCCAGAAGATACCACTT
CGCGATCCCAGCAATATCCCCGATCCGTATGTTAAGCTGTATCTGTTGCCTGGACGCACCAA
GGAGTCGAAACGCAAGACGAGCGTGATCAAGGACAACTGCAACCCCGTCTACGATGCATCC
TTTGAGTACCTGATTTCCATTGCCGAACTCAGGCAGACGGAACTGGAGGTGACGGTGTGCAC
CCAAAAGGGATTCCTATCCGGCGGTAGTCCCATCATTGGCATGGTAGGTACCCGAAAGCAAC
CCCTTAGTTACAGACACAGCGCGTACGTCCTTCGCATCCTTATGATTCCCAAGTACATATTCT
GCAAGAGTACAGTATATATAGGAAAGATATCCGGGTGAACTTCGgcgttgtcatcggcaatTTCgGTTT
TAGAGCTAGAAATAGCAAGTTAAAATAAGGCTAGTCCGTTATCAACTTGAAAAAGTGGCAC
CGAGTCGGTGCTTTTTTGCTCACCTGTGATTGCTCCTACTCAAATACAAAAACATCAAATTTT
CTGTCAATAAAGCATATTTATTTATATTTATTTTACAGGAAAGAATTCCTTTTAAAGTGTATT
TTAACCTATAATGAAAAACGATTAAAAAAAATACATAAAATAATTCGAAAATTTTTGAATAG
CCCAGGTTGATAAAAATTCATTTCATACGTTTTATAACTTATGCCCCTAAGTATTTTTTGACC
ATAGTGTTTCAATTCTACATTAATTTTACAGAGTAGAATGAAACGCCACCTACTCAGCCAAG
AGGCGAAAAGGTTAGCTCGCCAAGCAGAGAGGGCGCCAGTGCTCACTACTTTTTATAATTCT
CAACTTCTTTTTCCAGACTCAGTTCGTATATATAGACCTATTTTCAATTTAACGTCGGTTTTGG
ACACTGGAACCGGTTTTAGAGCTAGAAATAGCAAGTTAAAATAAGGCTAGTCCGTTATCAAC
TTGAAAAAGTGGCACCGAGTCGGTGCTTTTTTGCCTACCTGGAGCCTGAGAGTTGTTCAATA
AAATAAAAATGTTTCGTTTTTTTGCTTTCGCCAGTATTTATTATTTTTCATCAATATGTATTCA
ATTTGGTATGTATTTAGTAATTGTAATATATAGACAATGGTTTTCCGTTGACGTACATACATC
TGACGTGTGTTTATTTAGACATAATAGTTATGTTTTCACATCTTTTTAATGTTCGCTTAATGCG
TATGCATTCTAGACAATTGTGCTCGGCAACAGTATATTTGTGGTGTGCCAACCAACAACCTG
CAGGAGCTCCAGCTTTTGTgctagcGTACGCGTATCGATAAGCTTtaaGATACATTGATGAGTTTG
GACAAACCACAACTAGAATGCAGTGAAAAAAATGCtttatttGTGAAATTTGTGATGCTATTGCttt
atttGTAACCATTATAAGCTGCAATAAACAAGTTAACAACAACAATTGCATTCATTTTATGTTTC
AGGTTCAGGGGGAGGTGTGGGAGGTTTTTTAAAGCAAGTAAAACCTCTACAAATGTGGTAT

GGCTGATTATGATCTAGAGTCGCggcccCTACAGGAACAGGTGGTGGCGGCCCTCGGCGCGCT
CGTACTGCTCCACGATGGTGTAGTCCTCGTTGTGGGAGGTGATGTCCAGCTTGGAGTCCACG
TAGTAGTAGCCGGGCAGCTGCACGGGCTTCTTGGCCATGTAGATGGACTTGAACTCCACCAG
GTAGTGGCCGCCGTCCTTCAGCTTCAGGGCCTTGTGGATCTCGCCCTTCAGCACGCCGTCGC
GGGGGTACAGGCGCTCGGTGGAcGCCTCCCAGCCCATAGTCTTCTTCTGCATTACGGGGCCG
TCGGAGGGGAAGTTCACGCCGATGAACTTCACCTTGTAGATGAAGGAGCCGTCCTGgAGGGA
GGAGTCCTGGGTCACGGTCACCACGCCGCCGTCCTCGAAGTTCATCACGCGCTCCCACTTGA
AGCCCTCGGGGAAGGACAGCTTCTTGTAGTCGGGGATGTCGGCGGGGTGCTTCACGTACACC
TTGGAGCCGTACTGGAACTGGGGGGACAGGATGTCCCAGGCGAAGGGCAGGGGGCCGCCCT
TGGTCACCTTCAGCTTGGCGGTCTGGGTGCCCTCGTAGGGGCGGCCCTCGCCCTCGCCCTCG
ATCTCGAACTCGTGGCCGTTCACGGAGCCCTCCATGCGCACCTTGAAGCGCATGAACTCCTT
GATGACGTCCTCGGAGGAGGCcatGGTGGCGACCGGCTTCGAGCCGATTGTTTAGCTTGTTCA
GCTGCGCTTGTTTATTTGCTTAGCTTTCGCTTAGCGACGTGTTCACTTTGCTTGTTTGAATTGA
ATTGTCGCTCCGTAGACGAAGCGCCTCTATTTATACTCCGGCGGTCGAGGGTTCGAAATCGA
TAAGCTTGGATCCTAATTGAATTAGCTCTAATTGAATTAGTCTCTAATTGAATTAGATCCCCG
GGCGAGCTCGgtcgaCCGTGGGCATCGGCAATACCACCactaatccgtgcccctatgcggtaaatgtctttgacttgacca
cggatacgcgaattcggagatacgagctacctggcgtggacacaaatccaaatactttcatagctaacattgccgtggatataggcaaaaattgcgatgat
gcatatgcctattttgccgatgaattgggatacggcttgattgcttactcctgggaactgaacaagtcctggagattctcggcacattcgtatttttccccgat
ccattgaggggcgatttcaatgtcgctggtattaacttccaatggggcgaggagggtatatttggtatgtccctttcgcccattcgatcggatggttatcgtac
cctgtactttagtccgttagcaagtcatcgacaatttgccgtatccacgaggattttgagggatgaaaccaggacggaagatagctatcatgactttgttgcc
ttagatgaacggggtccaaactcccataccacttcacgtgtgatgagcgatgatggaattgagctgttcaatttaatagatcaaaatgcagtgggttgctgg
cactcatcaatgccgtactcaccgcaatttcatggcattgtggatcgcgatgacgttggcttagtttttccggccgatgtgaaaattgatgagaacaaaaacg
tttgggttctatccgataggatgcccgttttcttgctgtctgacttggattattcagatactaatttccgaatttacacggctcccttggccactttaattgagaata
ctgtgtgtgatttgaggaataacgcctatgggccgccaaataccgtttcaataccaaaacaagccgttttgccaatgggtccaccgttatatacgaaacaat
atcgtcctgtcttgccacagaaacctcagaccagctgggcttcctcgccgcctcctccaagtcgcacttatttgcccgccaattcaggcaatgtagtctcca
gAAGGGCGAATTCCAGCACACTGGCGGCCGTTACTAGTGGATCCGAGCTCGGTACCAAGCTT
GATGCATAGCTTGAGTATTCTATAGTGTCACCTAAATAGCTTGGCGTAATCATGGTCATAGC
TGTTTCCTGTGTGAAATTGTTATCCGCTCACAATTCCACACAACATACGAGCCGGAAGCATA
AAGTGTAAAGCCTGGGGTGCCTAATGAGTGAGCTAACTCACATTAATTGCGTTGCGCTCACT
GCCCGCTTTCCAGTCGGGAAACCTGTCGTGCCAGCTGCATTAATGAATCGGCCAACGCGCGG
GGAGAGGCGGT
